# An Empathy and Arts Curriculum During a Pediatrics Clerkship: Impact on Student Empathy and Behavior

**DOI:** 10.15766/mep_2374-8265.11414

**Published:** 2024-07-12

**Authors:** Maya Neeley, Lealani Mae Y. Acosta, Mario Davidson, Charlene Dewey

**Affiliations:** 1 Associate Professor, Department of Pediatrics, Vanderbilt University Medical Center; 2 Associate Professor, Department of Neurology, Vanderbilt University Medical Center; 3 Associate Professor, Department of Biostatistics, Vanderbilt University Medical Center; 4 Professor, Department of Medicine, and Director, Educator Development Program, Vanderbilt University Medical Center; † Co-second author

**Keywords:** Arts Observation, Empathy, Pediatric Clerkships, Humanities (Art, Literature, Music), Pediatrics, Physician-Patient Relationship

## Abstract

**Introduction:**

Empathy is critical within medicine and improves patient outcomes and satisfaction. Empathy declines during the clerkship years due to the hidden curriculum, where students observe emotional distancing and desensitization by providers. Studies show arts curricula can preserve empathy but are limited by sample bias and preclerkship occurrence. We implemented and evaluated a brief pediatric clerkship arts curriculum to improve empathic behaviors.

**Methods:**

We created two 1-hour required small-group sessions for pediatric clerkship medical students. The first session paired arts observation techniques with various paintings. The students then applied these techniques to video-based simulated patient interactions in the second session. We used the Toronto Empathy Questionnaire (TEQ) and an empathy behavior checklist (EBC) as pre/post assessments to gauge self-reported empathy and empathetic behaviors. We compared responses of learners who attended the sessions (curriculum group) to learners unable to attend (control group).

**Results:**

Thirty-four students participated in the curriculum; 19 were in the control group. Neither the control nor the curriculum group had a significant change in pre/post TEQ scores. Students with pre-TEQ scores less than 45 who participated in the curriculum had significant improvement in post-TEQ scores compared to their control group counterparts (*p* = .02). On the EBC, there was a significant difference between the curriculum and control groups for those who explored more about the child/family's experience (*p* < .05).

**Discussion:**

Our work suggests that a brief clerkship arts curriculum is useful for improving self-reported empathy ratings and empathetic skills, particularly for students identified as having below-average empathy.

## Educational Objectives

By the end of this activity, learners will be able to:

1. Apply four strategies for arts observation to visual arts media.
2. Explore responses, including actions, dialogue, and behaviors, that convey empathy at the bedside.
3. Analyze simulated physician-patient encounters using learned observation techniques.
4. Propose empathetic responses that could enhance simulated physician-patient encounters.

## Introduction

Patients and doctors concur that empathy is one of the cornerstones of patient care.^1,2^ Empathy is defined as an ability to recognize and be sympathetic to the emotional states of others, often including a desire to support their well-being.^3^ It is displayed through verbal and nonverbal communication and indicates emotional engagement with the patient.^4^ Empathy within medicine promotes patient satisfaction and adherence to recommendations, as well as improving clinical outcomes.^5^ In addition, when patients perceive their doctors to be empathetic, they have decreased anxiety and lower emotional distress.^6^ However despite the benefits of an empathetic encounter, doctors frequently overlook empathetic opportunities.^7^

The clerkship years contribute significantly to students’ professional identity development and, in turn, to how they interact with and respond to their patients, colleagues, and the medical system as a whole.^8^ Much of what is practiced by attendings was picked up and honed as beginning learners. Multiple studies have shown a decline in empathy throughout medical school, which is hypothesized to arise primarily through the hidden curriculum students are exposed to during their clinical rotations, where they see emotional distancing and desensitization by medical providers.^9,10^ However, studies have also shown that educational interventions can be successful in both sustaining and improving empathy in medical students.^11^

One such intervention involves incorporating the humanities into medical school curricula. The landmark 2018 book *The Integration of the Humanities and Arts With Sciences, Engineering, and Medicine in Higher Education: Branches From the Same Tree*^12^ noted how the humanities help teach skills such as appreciation for context, analysis of relationships, and the importance of perspective. Each of these can enhance the framework for understanding a patient and encourage greater reflection within the clinical encounter. Many medical schools now offer longitudinal arts courses where engagement with visual media enhances self-reported empathy on validated empathy questionnaires.^13–15^ However, these courses, such as the 4-week elective described by Razael and colleagues,^16^ are limited by selection bias (students choose if they wish to participate), by occurrence in the preclinical years (before exposure to the hidden curriculum), and by a lack of explicit focus on transferring skills to the clinical setting. In our literature review, we were unable to find any required arts curriculum specifically targeting the clerkship year, with a concentration on purposeful applicability to the clinical setting.

In an effort to support student empathy throughout the clinical years, we implemented and evaluated a 2-hour empathy and visual arts curriculum (E&AC) during the pediatrics clerkship. The aim of the E&AC was to teach, underscore, and nurture empathy within clinical medicine by highlighting observation skills to identify patient emotions and discussing actions, dialogue, and behavior that could improve empathic student behaviors within the clinical setting. Our goal was to enhance empathy and reduce empathy decline during clinical training years.

## Methods

### Curriculum Development

The development team consisted of new and established curriculum designers; experts on empathy and the arts and humanities; board-certified physicians in pediatrics, neurology, and internal medicine; and a biostatistician. One member of the team held a master's degree specializing in curriculum design and evaluation.

To develop the E&AC, we followed Oliva's model for curriculum designing and created a logic model to outline program impact.^17^ After conducting a review of the literature and existing empathy tools, we assessed the needs of learners at our institution. Using Decety and Jackson's work on empathy as a foundation,^18^ we created two sequential weekly group-learning sessions led by a faculty facilitator (Appendices A–D). Decety and Jackson suggest that there are three collaborative components to empathy—emotion recognition (observing emotions through expressions/speech/behavior), perspective taking (understanding the perspective of another while remaining separate from them), and affective response (providing an appropriate emotional reply). We created (writing the case scenario and dialogue, directing, videotaping, and editing) three simulated video encounters using standardized patient actors through Vanderbilt University Medical Center's Center for Experiential Learning and Assessment (Appendices E–G). We designed the E&AC sessions to align learner objectives with assessment measures and evaluations (Appendices H–L) following the New World Kirkpatrick model for program evaluation.^19^

### Facilitators

Facilitators were physicians involved in direct patient care. Each had experience in small-group facilitation. As preparation, they familiarized themselves with Decety and Jackson's framework of empathy,^18^ the four visual strategies discussed in the sessions (see Implementation, below), and session materials. A detailed facilitator guide (Appendices B and D) was available for each session.

### Learners

The Vanderbilt University Medical Center pediatrics clerkship consists of an 8-week clinical training block. There are five blocks in each academic year, beginning in August. Each block has approximately 20 second-year medical students who have completed a foundational year of medical knowledge acquisition. On their clerkship, students engage in a variety of clinical learning opportunities within the hospital and also spend 2 weeks at a community pediatric practice. Our curriculum began in block 1 (August 2021) and ran through block 3 (March 2022); further sessions were stopped because of in-person restrictions related to the COVID-19 pandemic. The two 1-hour E&AC sessions were required for all students present on the pediatrics clerkship who were on a clinical rotation within the hospital.

Students on the same clerkship block who had a conflicting requirement within the medical school during the allotted time constituted a control group. Student participation in the curriculum and completion of surveys were not used for grading purposes.

### Setting

Each of the two E&AC sessions took place during the lunch hour when students were relieved of their clinical duties. To ensure the curriculum was available to all clerkship students, the E&AC occurred twice during each block, once during weeks 3–4 (session 1 on week 3, session 2 on week 4) and once during weeks 5–6 (session 1 on week 5, session 2 on week 6). This allowed students to have the first and last 2 weeks of the block (weeks 1–2 and weeks 7–8) to complete their pre/post surveys. Most sessions took place in person in a conference room within the hospital; however, due to more stringent restrictions during the Omicron surge, block 3 was completed via Zoom.

### Implementation

In the first session (Appendix A), the facilitator led participants to reflect upon common emotions they had seen within their clerkships and reviewed Decety and Jackson's three components of empathy.^18^ The facilitator then reviewed four different arts observation strategies: (1) the Five Question Protocol,^20^ (2) visual thinking strategies,^21^ (3) inquiry-based looking,^22^ and (4) denotations/connotations. Students practiced applying each observation strategy to selected artworks.

In the second session (Appendix C), participants began by discussing empathetic practices they had witnessed or provided during their clinical work. Next, they watched three videos of simulated physician-patient interactions (Appendices E–G) and utilized learned observation strategies to identify emotions seen in each interaction. The facilitator asked the students to assess how empathetic eash encounter was according to a milestone-like rating scale (Appendix H). The facilitator then led a discussion on what the physician in the video did well to convey empathy and what verbal and nonverbal behaviors, dialogue, and actions could have been used to further convey empathy.

At the end of each session, the facilitator distributed paper-based anonymous evaluation forms for the participants to complete (Appendices I and J). The evaluations contained both Likert-scale items quantitatively assessing student reactions to course content and free-text questions qualitatively assessing the curriculum's strengths and weaknesses and participants’ intent to change.

### Learner Assessment

We assessed learners using three main tools: (1) the Toronto Empathy Questionnaire (TEQ; Appendix K),^23^ (2) the Empathy Behavior Checklist (EBC)–Student (Appendix L), and (3) the EBC–Patient and Family (Appendix L). The TEQ consisted of 16 validated questions for empathy self-assessment rated on a 5-point scale (1 = *never,* 5 = *always*). Scores varied from 0 to 64, with those above 45 indicative of above-average empathy. Both the EBC–Student and EBC–Patient and Family were created by the authors utilizing the NURSE (name, understand, respect, support, explore) technique^24^ along with guidance from expert physicians across a variety of subspecialties on which behaviors best conveyed empathy in the clinical setting. The EBC–Patient and Family was reviewed by a hospital program manager specializing in patient education to ensure that terminology was appropriate for a fifth-grade reading level. The EBCs consisted of itemized behaviors that lent themselves to empathic interactions scored on a 3-point rating scale (1 = *not at all*, 3 = *often*), followed by an overall empathy rating and a free-text question.

We asked all clerkship students (both curriculum and control groups) to complete the TEQ and the EBC at two distinct points in time: once over the beginning 2 weeks of the clerkship block and once during the last 2 weeks of the clerkship block (Figure). Following a patient encounter, students were asked to complete an EBC–Student for themselves and to give the patient or family a QR code to access the EBC–Patient and Family online. All evaluations were administered using REDCap (research electronic data capture), a secure web-based software platform developed by Vanderbilt University,^25,26^ but any online software or paper-based system could be used. All data were collected using unique identifiers so that individual data could be grouped by person while still maintaining anonymity.

**Figure.**
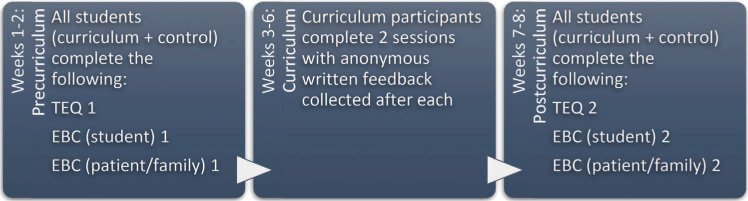
Timing of collection of survey materials by clerkship week. EBC = Empathy Behavior Checklist; TEQ = Toronto Empathy Questionnaire.

In sum, we assessed students’ reactions (session evaluation forms [Appendices I and J), learning (pre/post TEQ changes [Appendix K]), changes to behavior (pre/post self-assessment on the EBC–Student [Appendix L]), and potential improvements in patient care (pre/post patient/family assessment on the EBC–Patient and Family [Appendix L]).

### Data Analysis

To analyze the quantitative data collected, we calculated descriptive statistics and Wilcoxon rank sum tests on the pre/post deltas for the curriculum and control group students. We calculated signed rank tests on the pre/post TEQ. All analysis was done using R (version 4.0.4). Free-text responses were thematically analyzed through a process of coding, common themes generation, and review for accurate naming.

### Institutional Review Board

This curriculum assessment (IRB #202541) was approved as exempt by the Vanderbilt University Medical Center Institutional Review Board.

## Results

A total of 58 students completed the three pediatric clerkship blocks. Thirty-four students were enrolled in the curriculum group, participating in both E&AC sessions, and 19 students were assigned to the control group. Five students completed only one of the two sessions and were therefore removed from statistical analysis.

### Session Evaluations

Of the 34 participants in the curriculum group, 31 (91%) completed the session 1 evaluation, and 34 (100%) completed the session 2 evaluation. Overwhelmingly, students agreed or strongly agreed that objectives were met (session 1 = 97%, session 2 = 100%; Table 1), that they learned helpful observation tools (session 1 = 91%, session 2 = 94%), and that they felt comfortable using skills clinically (session 1 = 88%, session 2 = 91%).

**Table 1.**
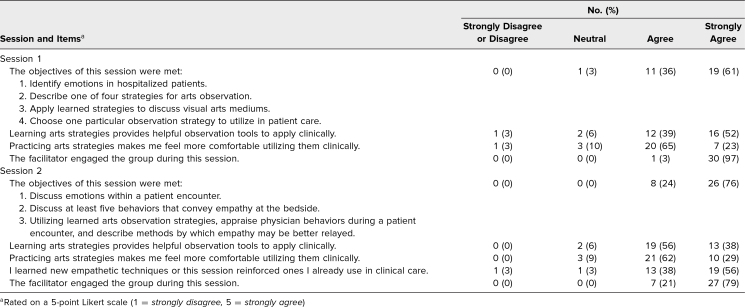
Summary of Student Evaluations of Sessions 1 (*N* = 31) and 2 (*N* = 34)

Before participating in the curriculum, in response to the session 1 question “Is there an observation strategy you already use that has helped you in clinical practice?”, 13 students (42%) reported not having an observation strategy, and 27 (79%) reported having empathetic strategies they used clinically. These mainly related to phrasing (e.g., “I'm sorry,” “Tell me more,” “This must be really hard”) and body language (e.g., eye contact, speaking while in a seated position, placing a hand on a shoulder). After session 1, in response to the session 2 question “Did you learn a new empathetic response in this session to use in the future? If so, what was it?”, all participants described strategies to use moving forward. These strategies covered three themes: attentiveness to context, looking for more, and ascribing meaning (Table 2).

**Table 2.**
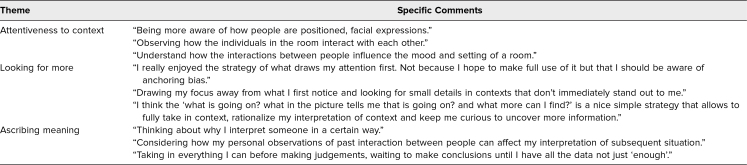
Clerkship Students’ Thematic Qualitative Responses on Observation Strategies for Discerning Empathetic Opportunities

After participating in the curriculum, in response to the session 2 question “Are there common empathetic responses you use in patient care?”, participants reported gaining new empathetic strategies including naming emotions (e.g., “It seems like you are feeling [emotion], can you tell me a little more about this?”), probing emotions (e.g., “Can we talk a moment about what is worrying you?”), and validation (e.g. “Telling parents that they are doing a good job, that they were justified in bringing their child to the hospital, and that the child's illness is not their fault”).

When asked “What from this session was most meaningful to you?”, students commented on the utility of hearing the varying perspectives of their peers, sharing their experiences, spending time discussing empathy and emotions, and the impact such sessions have on the physician-patient relationship. Responses included the following:

• “The most meaningful part of this was having time and an emphasis to reflect on and better my relationships with patients.”
• “Recognizing that empathy is something I can reflect on after any encounter I have. I have thought plenty about how I could improve my differential, exam, history, etc after a patient interaction, but asking myself how my empathy was during that visit could really help me become a better doctor.”

When asked “What about this session can be improved moving forwards?”, students appreciated “a lecture not talking about test subjects” and wished “we could have more creative sessions like this since we get pure medicine/science most of the time.” Students did report a history of Zoom fatigue and found it more difficult to interact over such a platform in block 3 when social distancing was mandated at our hospital.

### TEQ

Forty-seven students (81%) completed pre/post TEQs. Overall, no statistically significant differences were noted on the pre/post TEQs between curriculum and control groups. Seven students who completed pre/post TEQs (15%) had initial scores less than 45, indicating lower than average empathy. Within that subset of students, we found evidence of a significant difference in the pre/post TEQs between those in the curriculum group compared with those in the control group (*p* = .02). With 95% confidence, the difference was between 0.5 and 5.5 points.

### EBC

Twenty-eight (48%) students completed the pre/post EBC–Student. Sixteen (57%) belonged to the curriculum group and 12 (43%) to the control group. For most itemized behaviors and overall empathy score, we found no statistically significant difference on pre/post EBC results between the curriculum and control groups. However, we did find evidence that there was a difference on pre/post EBC deltas between the curriculum and control groups for those who self-reported exploring more about the child/family's experience (*p* < .05). Only three students completed the pre/post EBC–Patient and Family, so we were not able to complete statistical analysis on those surveys.

## Discussion

We created the E&AC as a curriculum focused on arts and empathy and geared toward the clerkship year. Our curriculum is notable because it was required, compared participants to a control group, and occurred within a medical student clerkship and during exposure to the hidden curriculum. In our literature review, we were unable to find another arts curriculum occurring within this specific context. Our curriculum is also unique in that we explicitly connect the skills garnered through arts observation directly to patient care examples, something that has been difficult to integrate in other studies.^16^

Per our literature review, the duration of most described arts programs is variable, with a range of 1.5–160 hours and an average of 18.4 hours.^27^ Because the pediatric rotation is already tightly scheduled, we needed to create a brief curriculum and chose to design two 1-hour interactive sessions drawing from existing literature on arts education to frame our work. The collaborative nature of the sessions allowed students to engage in dialogue together over the questions presented. These questions aimed to uncover students’ observations and provoke reflection on them. In addition, we needed to complete these sessions in the hospital so that students could promptly return to clinical care experiences. As a consequence, we were not able to partner with an art institution, unlike many other arts and humanities medical school courses.^14^ Fortunately, many paintings are available through open access, and we were able to select ones we felt would lead to thoughtful discussion based on the specific observation technique being taught. As a result, we successfully implemented an arts and humanities course without incurring expense or forming relationships with local art museums, which may not be present in the communities of many medical schools.

Our work demonstrates that an art-based curriculum can be implemented successfully during a pediatric clerkship and within the hospital amid clinical care responsibilities. These two sessions are easily adaptable to other institutions due to the format used. Minimal technology is required, and each session fits easily into a noon time slot. Based on feedback received, these sessions are most appreciated when held in person.

The students who benefited most from the curriculum were those with initial empathy scores less than 45. A statistically significant improvement was noted between pre- and post-TEQs for this subset of students. This suggests that students with below-average empathy scores may be those who benefit the most from such a targeted curriculum and that even a brief 2-hour curriculum can impact empathy in a significant manner. Despite students’ positive response to sessions and intent to change, there was no significant difference overall across pre/post TEQs between the curriculum and control groups. A ceiling effect may have confounded the data due to the high average pre-TEQ empathy score (54) across all students. These results may have also been confounded given that both sessions in block 3 were held virtually. Recent studies suggest that learning activities are less effective when presented through online as compared to in-person discussion formats.^28^ Finally, this curriculum occurred on a pediatrics clerkship; it is unclear if exposure to the hidden curriculum in this specialty is different from that in other specialties. We did not find a statistically significant decline in pre/post TEQ scores of students from the control group.

Regarding changes in behavior, curriculum group participants were more likely on the self-reported EBC–Student to explore more about the patient's and family's experience with their illness—a valuable step towards building an empathetic encounter. This exploration aligns with the cognitive component of empathy (perspective taking) and, along with the affective component (sharing in the emotional experience after such perspective taking), helps to build an empathetic encounter.^19^ A recently published study noted similar results for first-year medical students engaging in an arts program.^16^ It may be that the perspective-taking component of empathy is the skill most impacted by this type of curriculum.

Across Kirkpatrick's model for program evaluation,^19^ we gathered favorable feedback related to reaction, learning, and behavior (Kirkpatrick levels 1–3). There was an overwhelmingly positive response to both sessions in terms of content and influence. The majority of students felt that objectives were met, learned new observation techniques and empathetic responses, felt more comfortable utilizing these new skills clinically through small-group practice, and intended to use them in future clinical interactions. Many students commented on the E&AC's value; it was a unique humanities exercise within the clinical clerkship that allowed them space to share experiences, hear varying perspectives, and highlight the role of empathy in clinical practice and the physician-patient relationship. TEQ results suggest that a curriculum such as this can be targeted to students who may have less than average empathy or those whose empathic skills may be lower than their peers. EBC results suggest that those who participated in the curriculum did engage in new behaviors correlated with the perspective-taking aspect of empathy. Level 4 of Kirkpatrick's model was difficult to assess. Our rate of return on the pre/post EBC–Patient and Family was so low that minimal information could be gained about whether encounters with students improved after the curriculum.

Our curriculum evaluation is limited by sample size and survey return rate, which may have influenced statistical power. We had an overall positive response rate for the TEQ. Participants completed it more frequently than the EBC, possibly due to survey design and/or fatigue. Additionally, each of the surveys is limited by biases that can occur with self-evaluation; in order to best evaluate attitude and skill application, it would have been helpful to include family/guardian assessment. While we had hoped to do so, it was very difficult in a REDCap survey format, and so, another method of assessment may be indicated to improve completion rates. In addition, the curriculum is limited by participant number, duration, and exposure to the E&AC content. Our students are second-years and may have limited application of results when compared to students in their third- or fourth-year clinical rotations. We did not study the long-term effects to determine if the improvement in below-average TEQ scores would be maintained over the clinical training years.

Like any other skill, empathy can be practiced and enhanced.^29^ Our results suggest that a brief arts curriculum can improve self-reported empathy ratings and empathetic behaviors, particularly for those students identified as having less than average empathy. Expanding this curriculum to include multiple institutions and more participants, determining how to best obtain patient/family feedback, and completing follow-up surveys with participants would all be helpful to ascertain its long-term impact and full benefits to learners, patients, and families.

## Appendices


Empathy Session 1.pptxEmpathy Session 1 Facilitator Guide.docxEmpathy Session 2.pptxEmpathy Session 2 Facilitator Guide.docxEmpathy Video 1.mp4Empathy Video 2.mp4Empathy Video 3.mp4Empathy Session 2 Student Handout.docxEmpathy Session 1 Evaluation Form.docxEmpathy Session 2 Evaluation Form.docxToronto Empathy Questionnaire.docxEmpathy Behavior Checklists.docx
*All appendices are peer reviewed as integral parts of the Original Publication.*
