# Supplementary material for: An Empathy and Arts Curriculum During a Pediatrics Clerkship: Impact on Student Empathy and Behavior
Source: MedEdPORTAL. 2024 Jul 12;20:11414. doi: 10.15766/mep_2374-8265.11414 (PMC11239799; doi:10.15766/mep_2374-8265.11414)
Supplement: Supplementary file 1 — Empathy Session 1.pptxEmpathy Session 1 Facilitator Guide.docxEmpathy Session 2.pptxEmpathy Session 2 Facilitator Guide.docxEmpathy Video 1.mp4Empathy Video 2.mp4Empathy Video 3.mp4Empathy Session 2 Student Handout.docxEmpathy Session 1 Evaluation Form.docxEmpathy Session 2 Evaluation Form.docxToronto Empathy Questionnaire.docxEmpathy Behavior Checklists.docx [file mep_2374-8265.11414-s001.zip › B. Empathy Session 1 Facilitator Guide.docx]

**Empathy Session 1: Facilitator Guide/Outline**

| **Session Objectives** | **Session Timeline** |
| --- | --- |
| Identify emotions in hospitalized patients | 0-15min: Introduction/small group sharing/ground rules (welcome/review of objectives/review of empathy components/shared witnessed emotions) |
| Describe 1 of 4 strategies for arts observations | 15-55min: Review of observation techniques/small group discussion as applied to various paintings |
| Apply learned strategies to discuss visual arts mediums | 55-60min: Wrap up/conclusions |
| Choose one particular observation strategy to utilize in patient care |  |

**Introduction: (15 minutes) slides 1-4**

Let us quickly review our objectives for this time together. By the end of this session, we want to…

- Identify emotions in hospitalized patients
- Describe 1 of 4 strategies for arts observation
- Apply learned strategies to discuss visual arts mediums
- Choose one particular observation strategy to utilize in patient care

What is empathy? The ability to understand and share the feelings of others

Decity and Jackson say there are three components to empathy

1. Emotion recognition: the cognitive ability to infer what another is feeling
2. Perspective taking: the ability to experience another person’s emotions
3. Affective response: responding with compassion/concern

Today we’re going to talk about some strategies for parts 1 and 2: that emotional recognition and perspective taking. How do we build our observation skills to recognize emotions in our patients and see their perspectives?

Let’s talk about emotion for a minute. The hospital is filled with them! What have you seen so far?

*Common emotions to discuss: guilt, loneliness, stress/worry/anxiety, fear, annoyance, agitation, sadness, anger, disgust, exhaustion, surprise, hope, gratefulness*

How do you recognize them? Trying to learn how to figure out the story and then figuring out what disease the story is trying to tell you, that is a lot all by itself. And then we’re saying you also have to see the emotions behind the story and then try and address them?!

That’s even more!

So this first session that we have, we’re just going to talk about strategies to observe. We’re going to practice these skills on paintings but I think some of these techniques will be helpful to you as you see your patients. All of these techniques are a little bit similar and a little bit different but I feel they all promote this concept of slow looking, taking time to both observe and analyze something in front of you. And I hope that by the end, you will find one or a part of one that maybe you will try out over the next couple of weeks as you see patients.

So we’re going to have a couple of ground rules.

Please try and be engaged. Even if you’re not into this, please try to participate over the next hour.

Please share your thoughts and listen kindly to the thoughts of others.

There are no right or wrong answers here. There is just what you see and what that means or what that speaks, all through the lens of you and your observations.

**Small Group Facilitated Conversations: (each strategy 2 minutes, time for discussion 8 minutes – 40 minutes total)**

**Strategy 1: The Five Question Protocol (slides 5-8)**

- What do you see?
- Does this remind you of anything?
- What is the story?
- What information would confirm that story?
- What did you observe about yourself
  - What was it like to be intentionally slowed down?
  - What initially stood out to you? Why?
  - How did they respond when a colleague noticed something they had not seen?
  - How did they respond when a colleague had a different interpretation?

*About the painting: The title of this piece is “Corridor in the asylum.” As many of you may know, Vincent Van Gogh struggled for years with his mental health and he was admitted to this psychiatric hospital where he continued to paint. He died by suicide a few months later.*

*Does knowing the context change how you frame the piece? How do contextual factors influence what we observe and how we interpret it? How are our observations affected when it is a febrile neonate vs. a child w/a new diagnosis of lupus?*

**Strategy 2: Visual Thinking Strategies (slides 9-12)**

What is going on in this picture?

What do you see that makes you say that?

What more can we find?

*About the painting: The title of this piece is “The Death of Socrates” and was painted by Jacques Louis David. Socrates was convicted by the Athenian courts, he died willingly, accepting a drink of hemlock. The courts hated him but a number of his followers lamented his fortune.*

*Tell me a little more about how the interactions between people influenced what you interpreted from this piece. How does that influence your interpretations in the clinical setting?*

**Strategy 3: Inquiry-based looking (slides 13-16)**

What stands out most in this image? What makes it stand out to you?

What do you think the image communicates? Based on what you see, how do you know this?

*About the painting: This is “The Gulf Stream” by Winslow Homer, who was obsessed with water. The painting depicts a strong Atlantic current and a solitary man alone with much going on in the background (the storm approaching, sharks in the water, and a possible rescue ship approaching). The painting has been interpreted as an expression of the artist’s presumed sense of mortality and vulnerability.*

*What initially stood out to you? Why? How did you make yourself see more, beyond what stood out immediately?*

**Strategy 4: Denotations & Connotations (slides 17-20)**

What do I see? (do not ascribe meaning yet, just use descriptive language)

What does it mean? (now you can ascribe meaning)

*About the painting: This piece is entitled “Cape Cod Evening” and it was painted by Edward Hopper. Critics say that it signifies the loss of rural America and people left behind.*

*What might influence your interpretations of denotations?*

*Considerations include:*

*Prior experiences/knowledge*

*Current distractions*

*Fatigue*

**Wrap-up (5 minutes) (slide 21)**

Thank you all for your participation!

I hope that this time together was valuable to you.

We have been able to reflect upon what emotions you have witnessed in your clinical time. We have thought about observation strategies typically used for viewing art, and considered how other things might influence them and our interpretations from them. While they are used for viewing art, I would like you to consider taking them into the clinical setting – how can you see more during your time with patients? What might these observations mean as you consider patient emotions and how to respond/react to them?

I look forward to our time together next week where we will talk more about this.

Before you leave, I’m going to give you a little bit of time to complete these anonymous surveys of today’s session. Please just drop them face-down on the chair in the back of the room. (**slide 22)**
