# Supplementary material for: An Empathy and Arts Curriculum During a Pediatrics Clerkship: Impact on Student Empathy and Behavior
Source: MedEdPORTAL. 2024 Jul 12;20:11414. doi: 10.15766/mep_2374-8265.11414 (PMC11239799; doi:10.15766/mep_2374-8265.11414)
Supplement: Supplementary file 1 — Empathy Session 1.pptxEmpathy Session 1 Facilitator Guide.docxEmpathy Session 2.pptxEmpathy Session 2 Facilitator Guide.docxEmpathy Video 1.mp4Empathy Video 2.mp4Empathy Video 3.mp4Empathy Session 2 Student Handout.docxEmpathy Session 1 Evaluation Form.docxEmpathy Session 2 Evaluation Form.docxToronto Empathy Questionnaire.docxEmpathy Behavior Checklists.docx [file mep_2374-8265.11414-s001.zip › A. Empathy Session 1.pptx]

## Slide 1
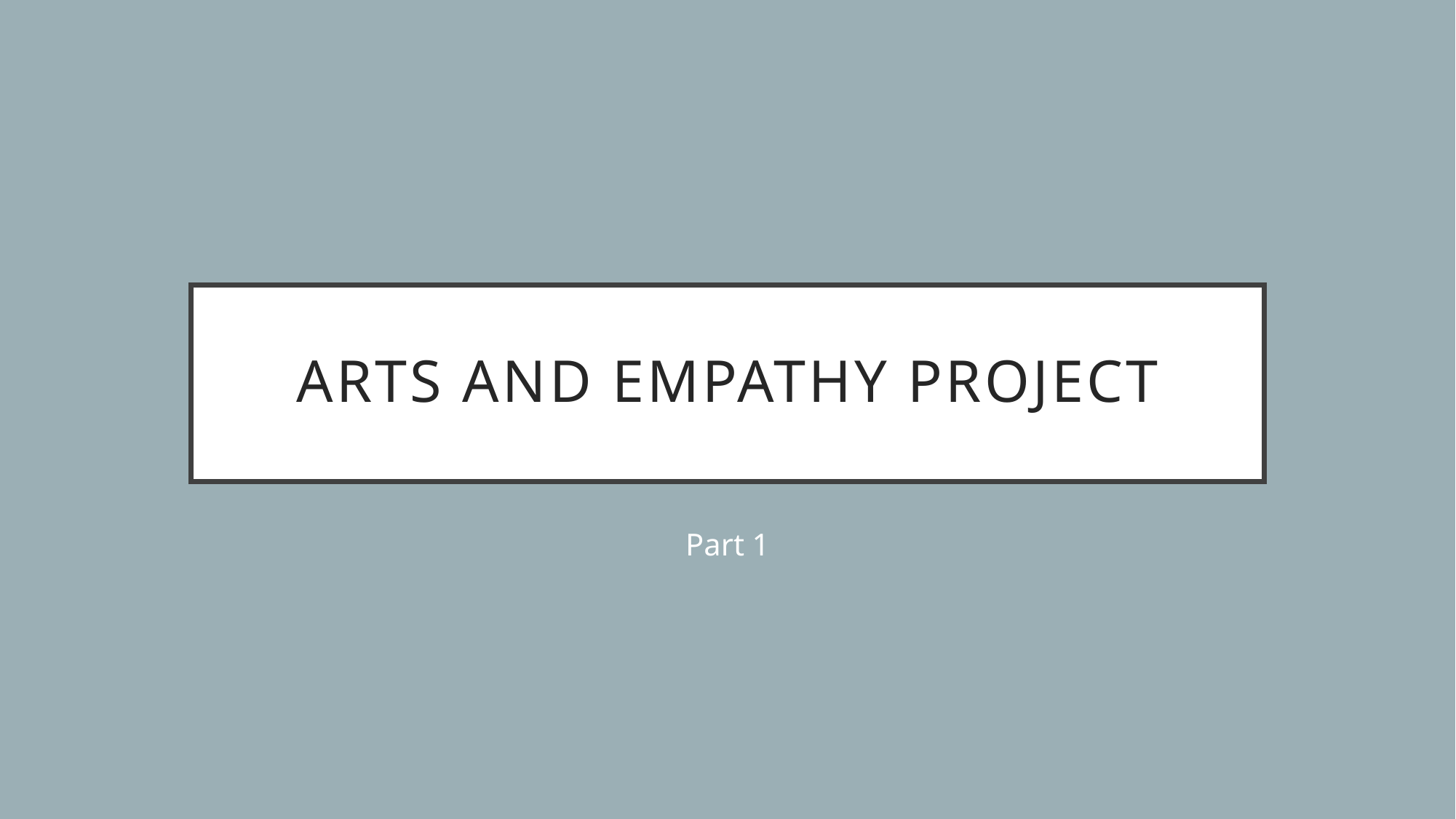

# Arts and Empathy Project
Part 1

## Slide 2
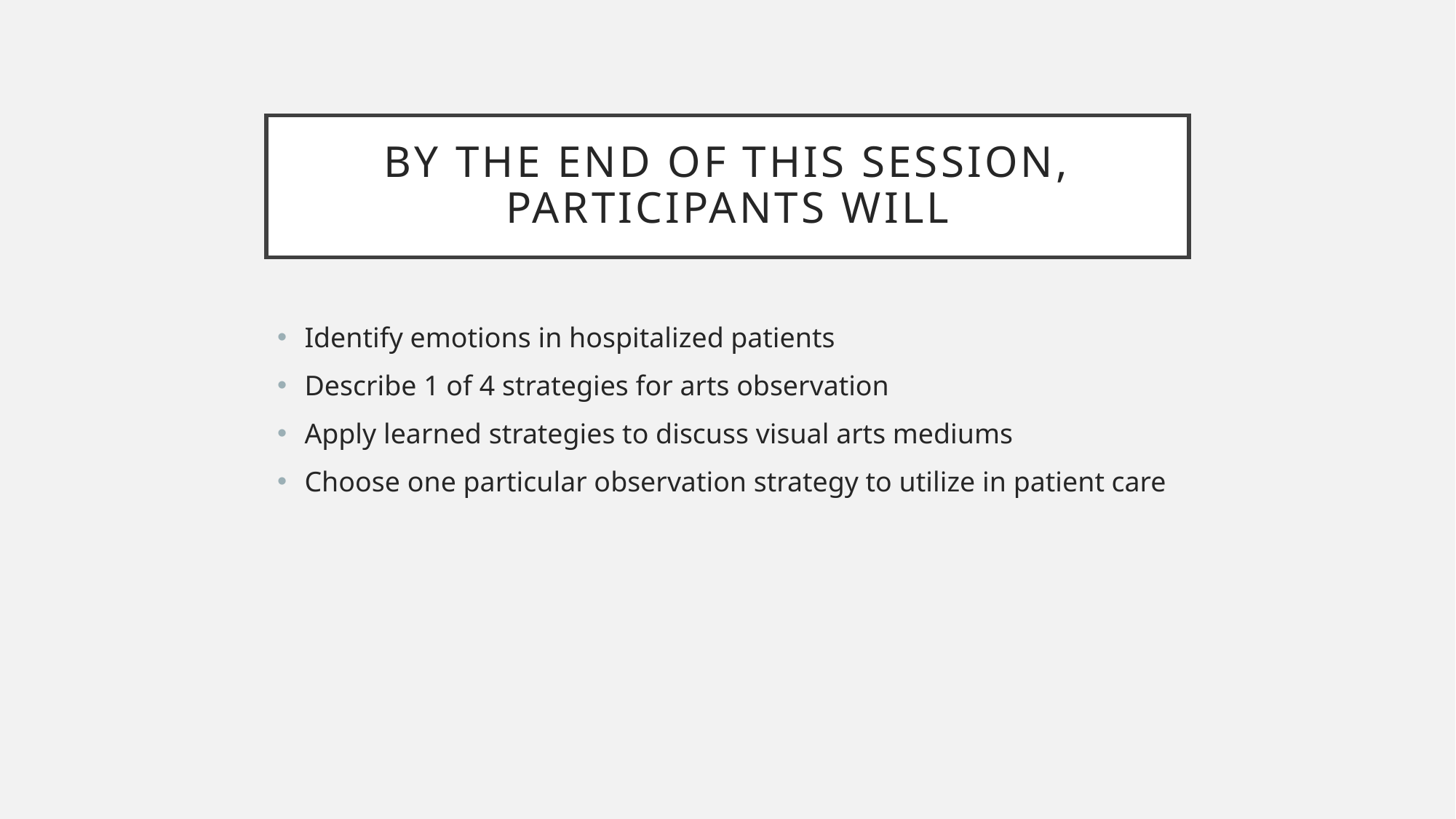

# By the end of this session, participants will
Identify emotions in hospitalized patients
Describe 1 of 4 strategies for arts observation
Apply learned strategies to discuss visual arts mediums
Choose one particular observation strategy to utilize in patient care

## Slide 3
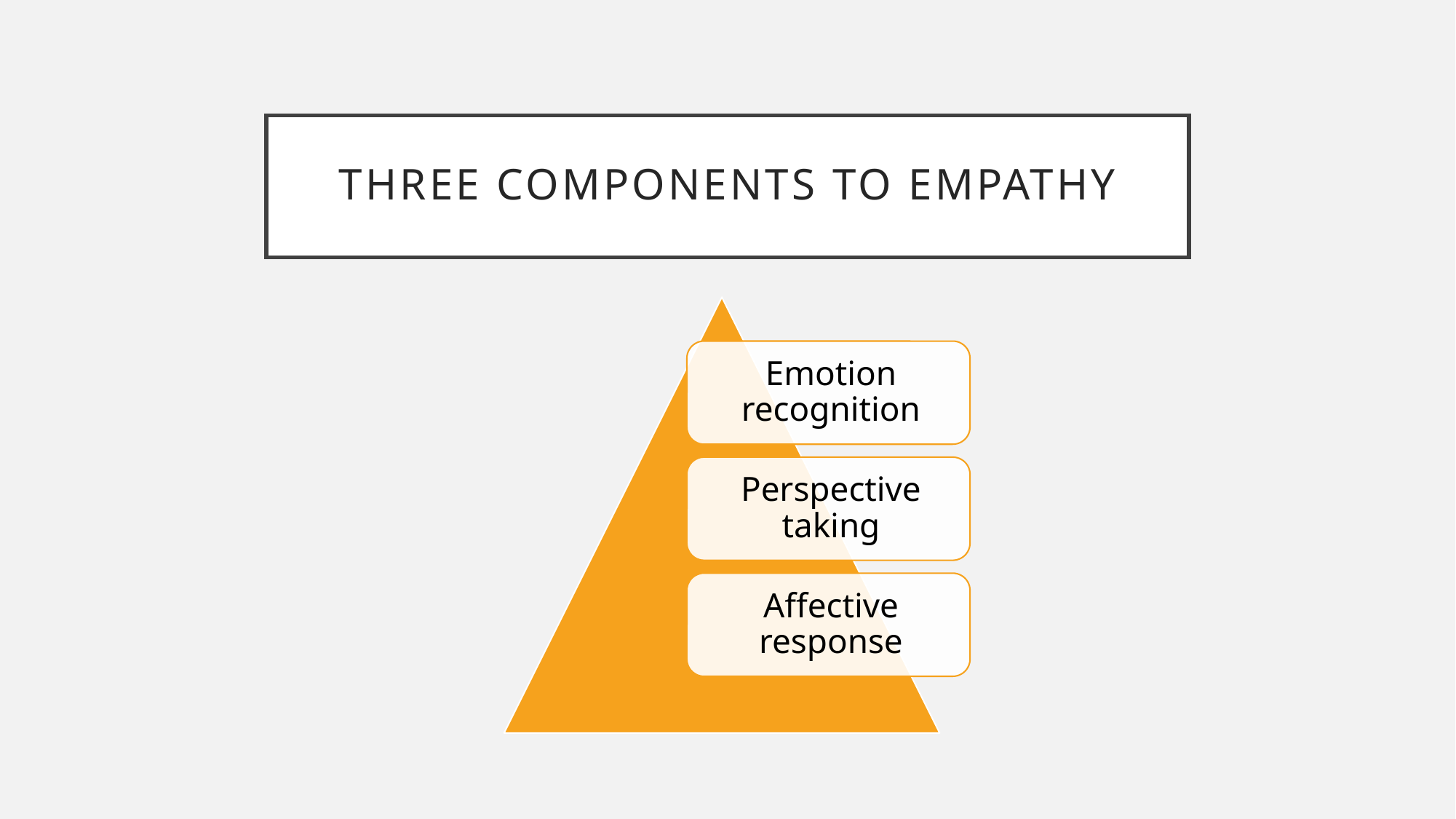

# Three components to empathy

## Slide 4
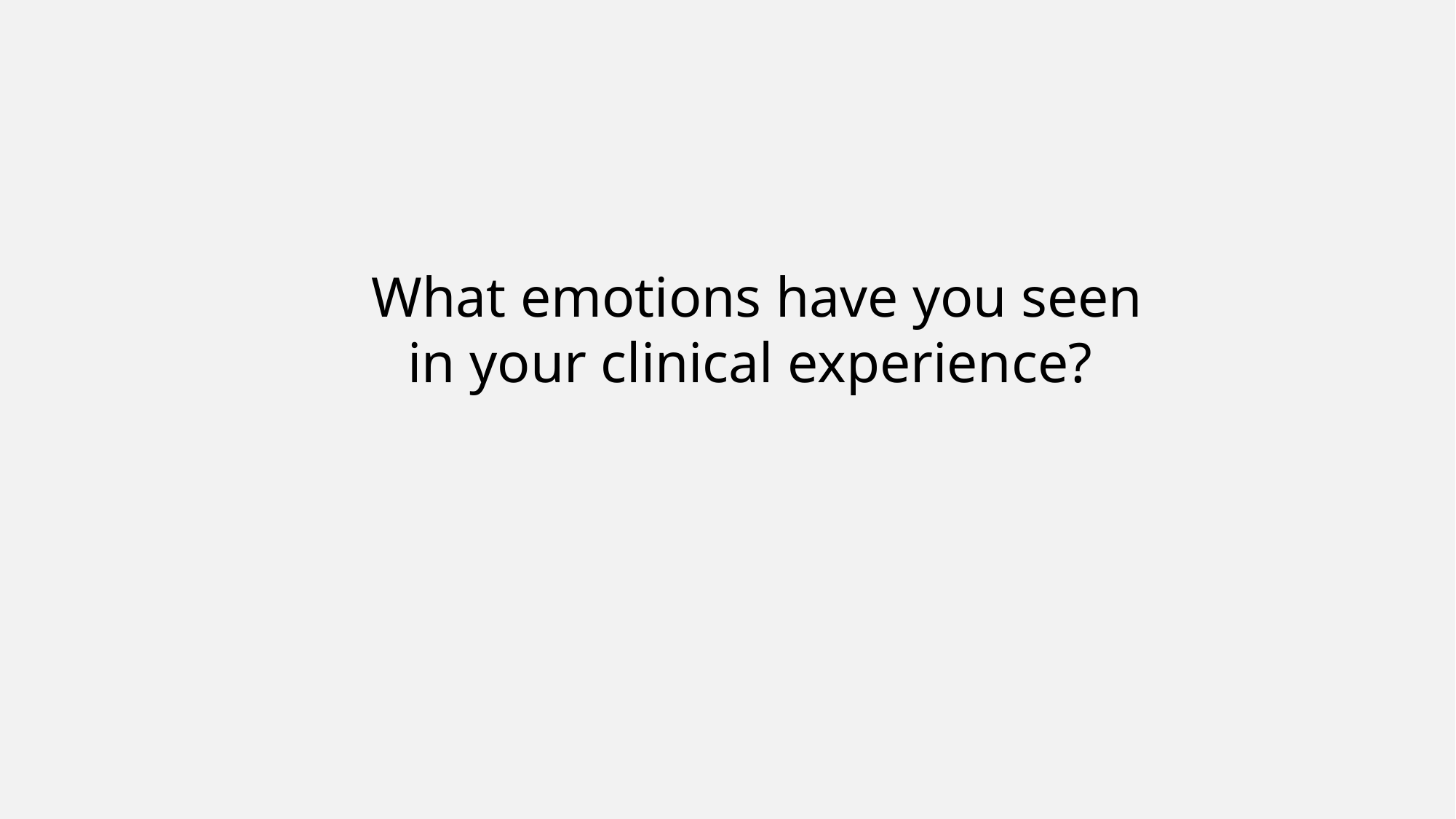

What emotions have you seen in your clinical experience?

## Slide 5
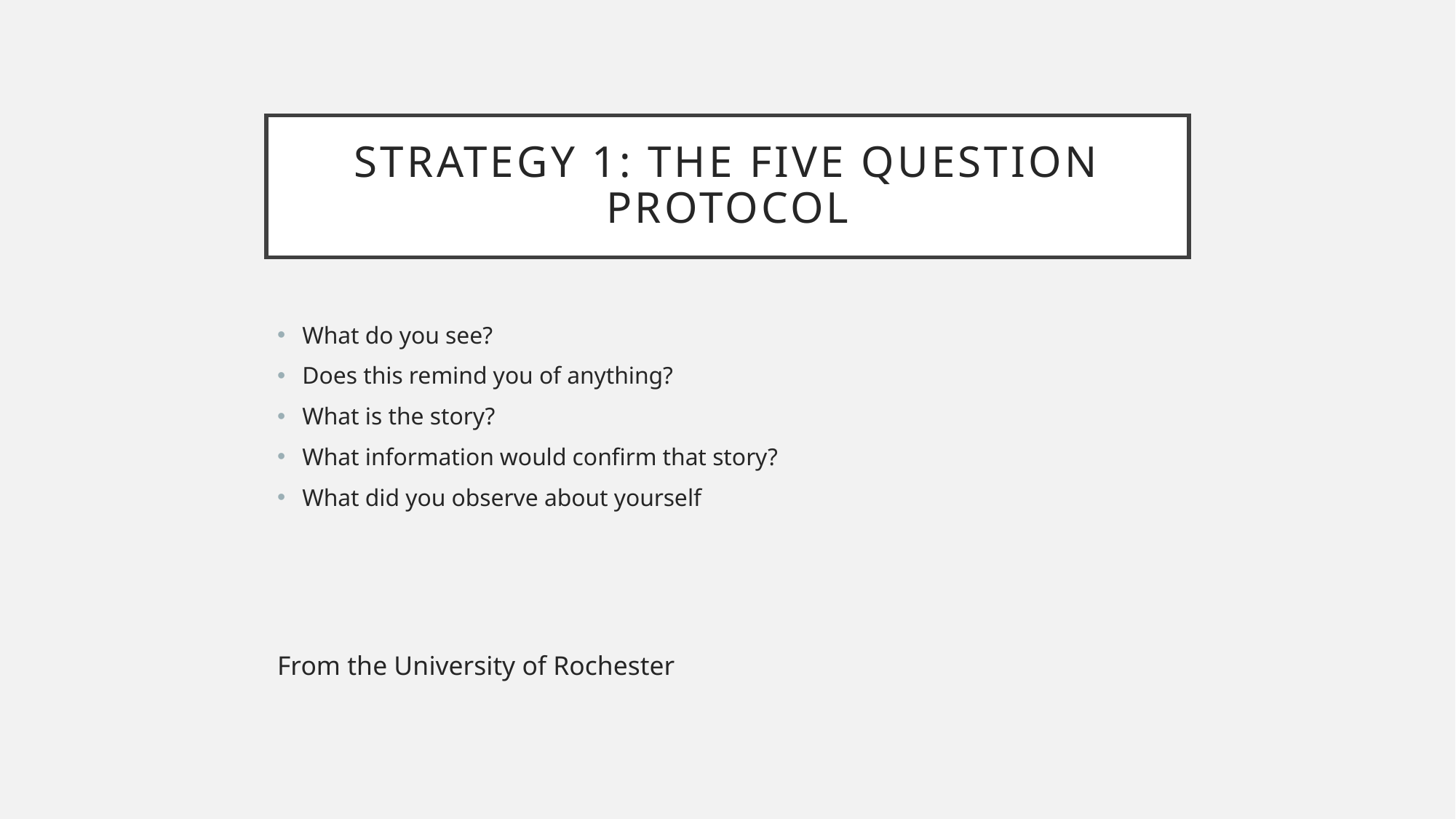

# Strategy 1: The Five Question Protocol
What do you see?
Does this remind you of anything?
What is the story?
What information would confirm that story?
What did you observe about yourself
From the University of Rochester

## Slide 6
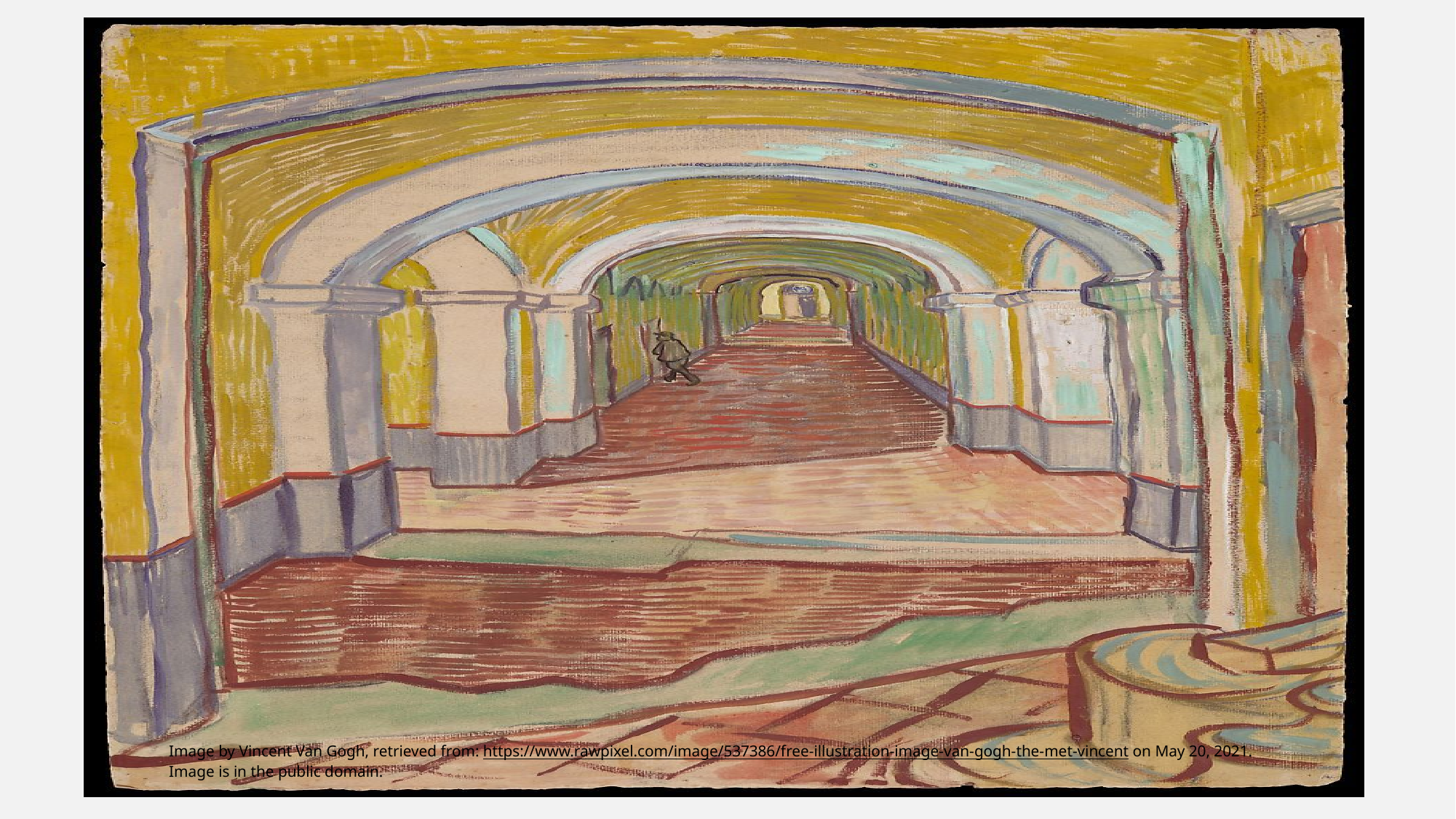

Image by Vincent Van Gogh, retrieved from: https://www.rawpixel.com/image/537386/free-illustration-image-van-gogh-the-met-vincent on May 20, 2021. Image is in the public domain.

## Slide 7
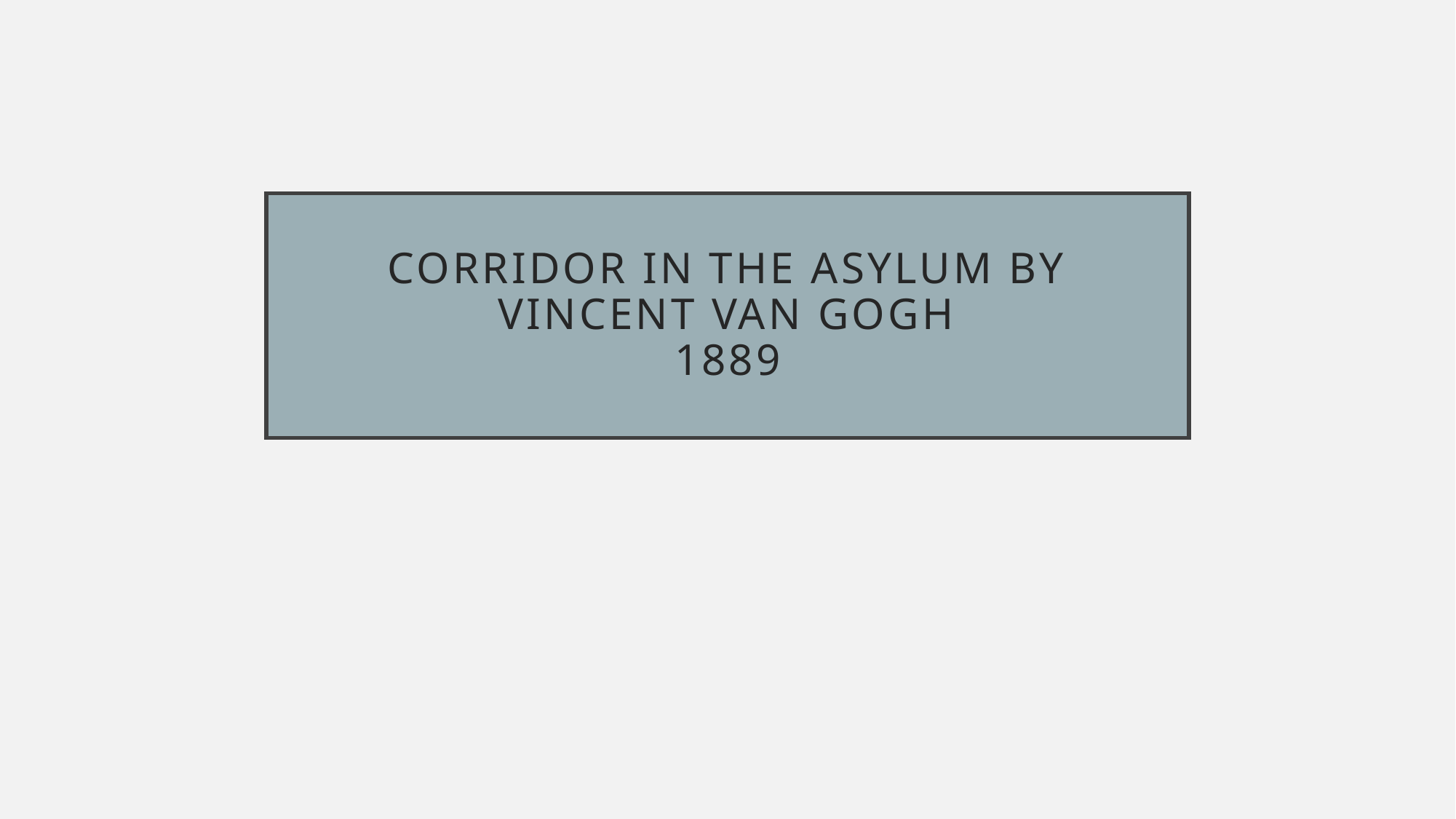

# Corridor in the asylum by Vincent van gogh 1889

## Slide 8
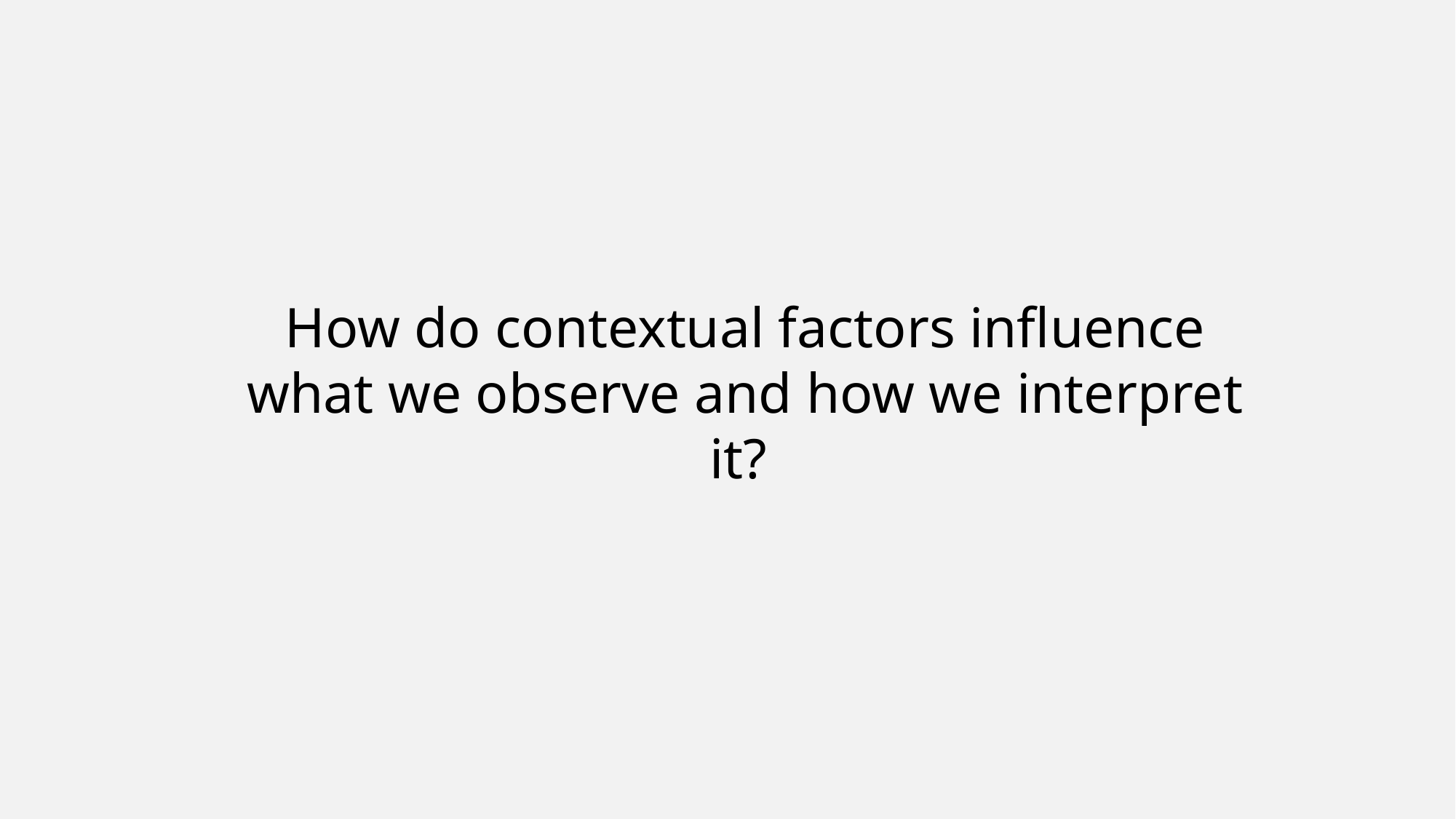

How do contextual factors influence what we observe and how we interpret it?

## Slide 9
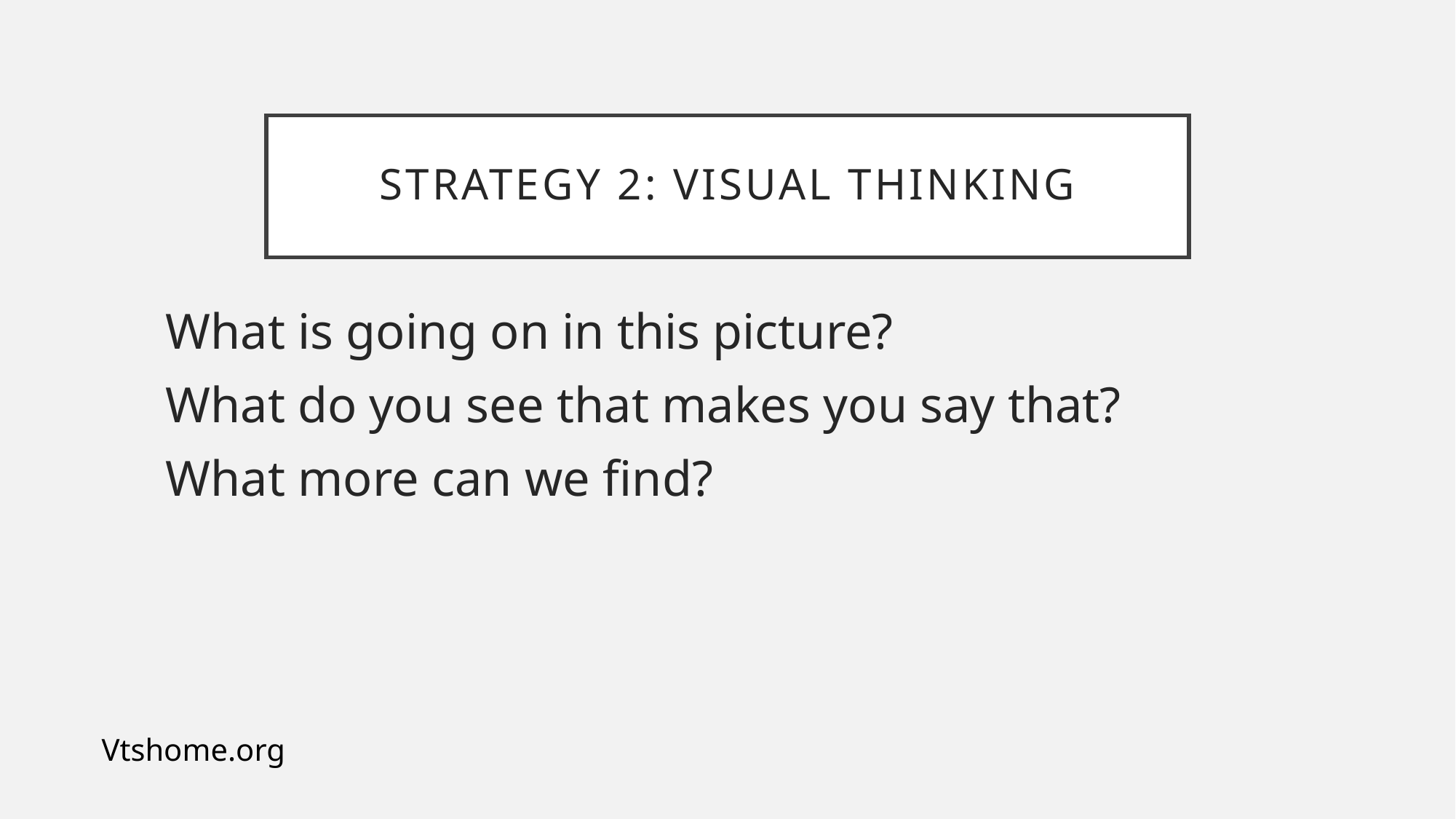

# Strategy 2: Visual Thinking
What is going on in this picture?
What do you see that makes you say that?
What more can we find?
Vtshome.org

## Slide 10
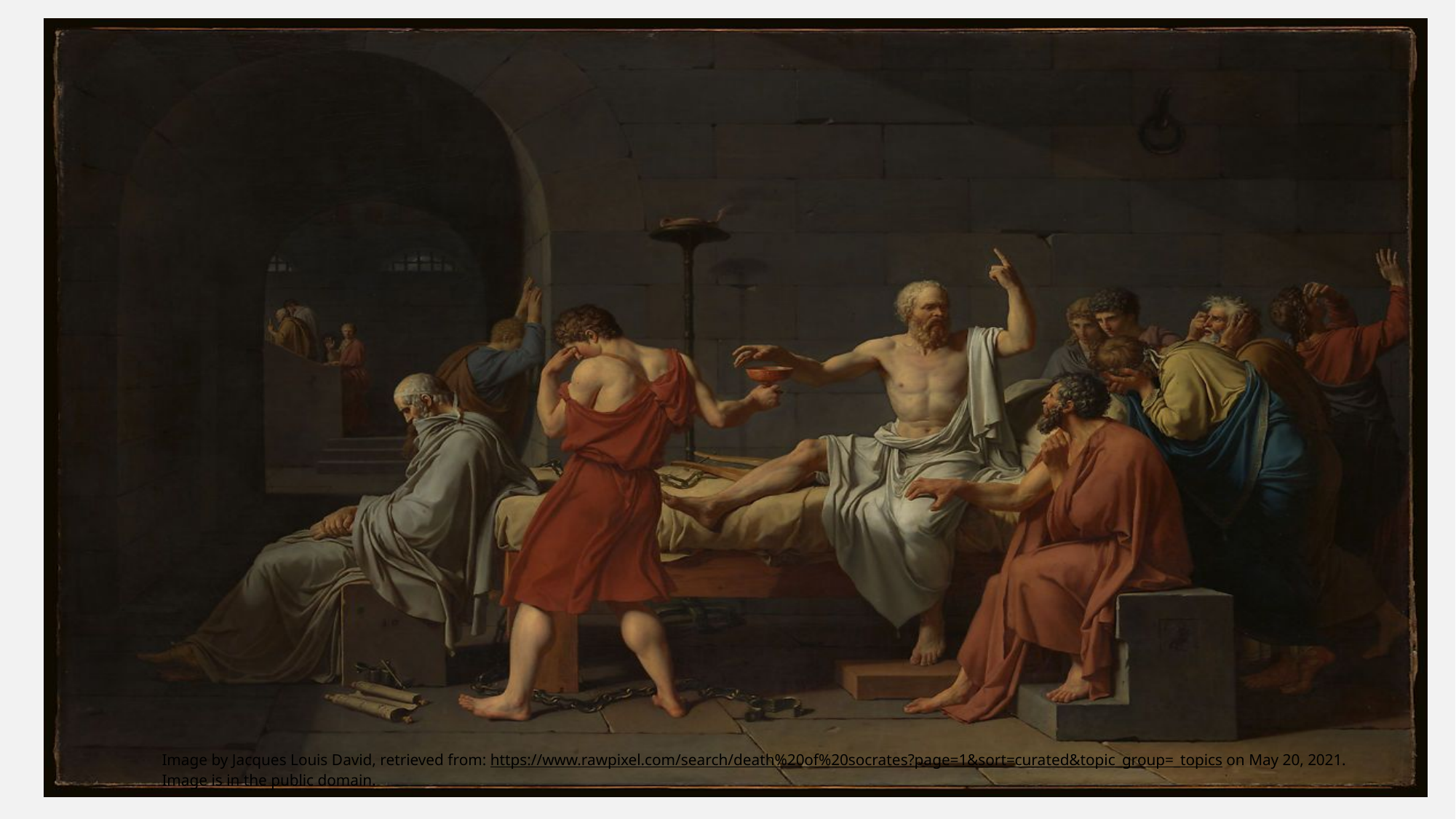

Image by Jacques Louis David, retrieved from: https://www.rawpixel.com/search/death%20of%20socrates?page=1&sort=curated&topic_group=_topics on May 20, 2021. Image is in the public domain.

## Slide 11
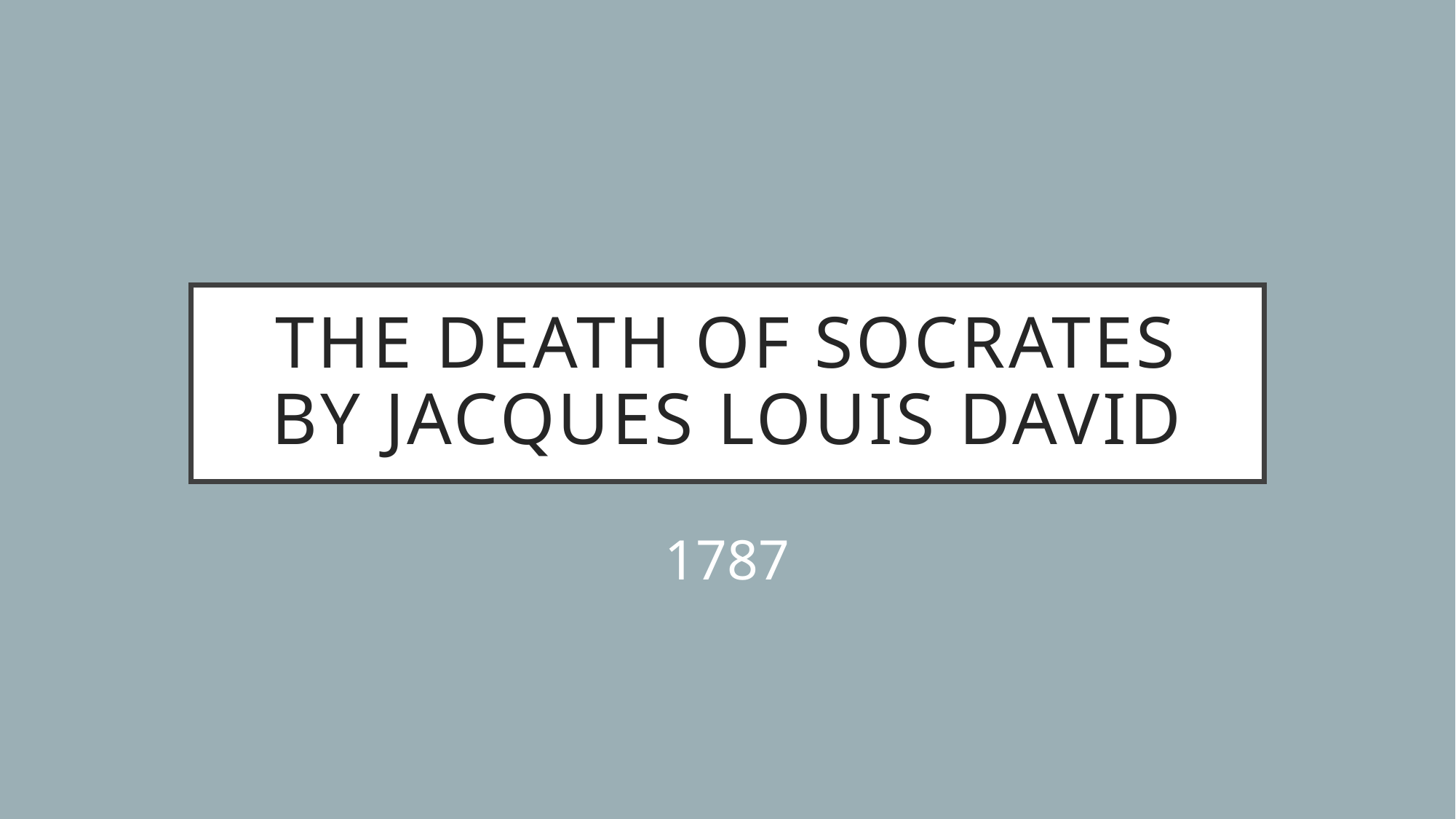

# The Death of Socrates by Jacques Louis David
1787

## Slide 12
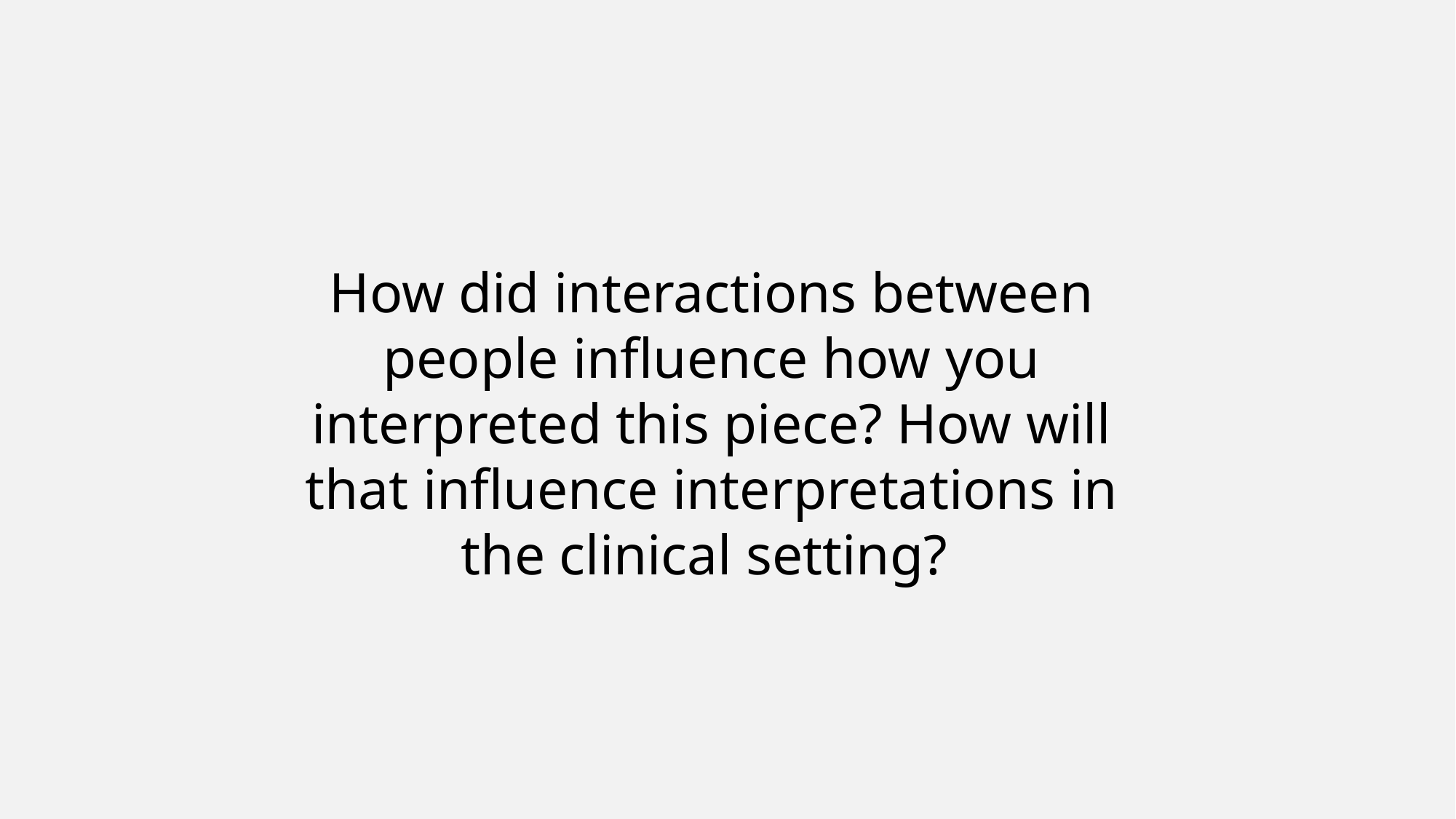

How did interactions between people influence how you interpreted this piece? How will that influence interpretations in the clinical setting?

## Slide 13
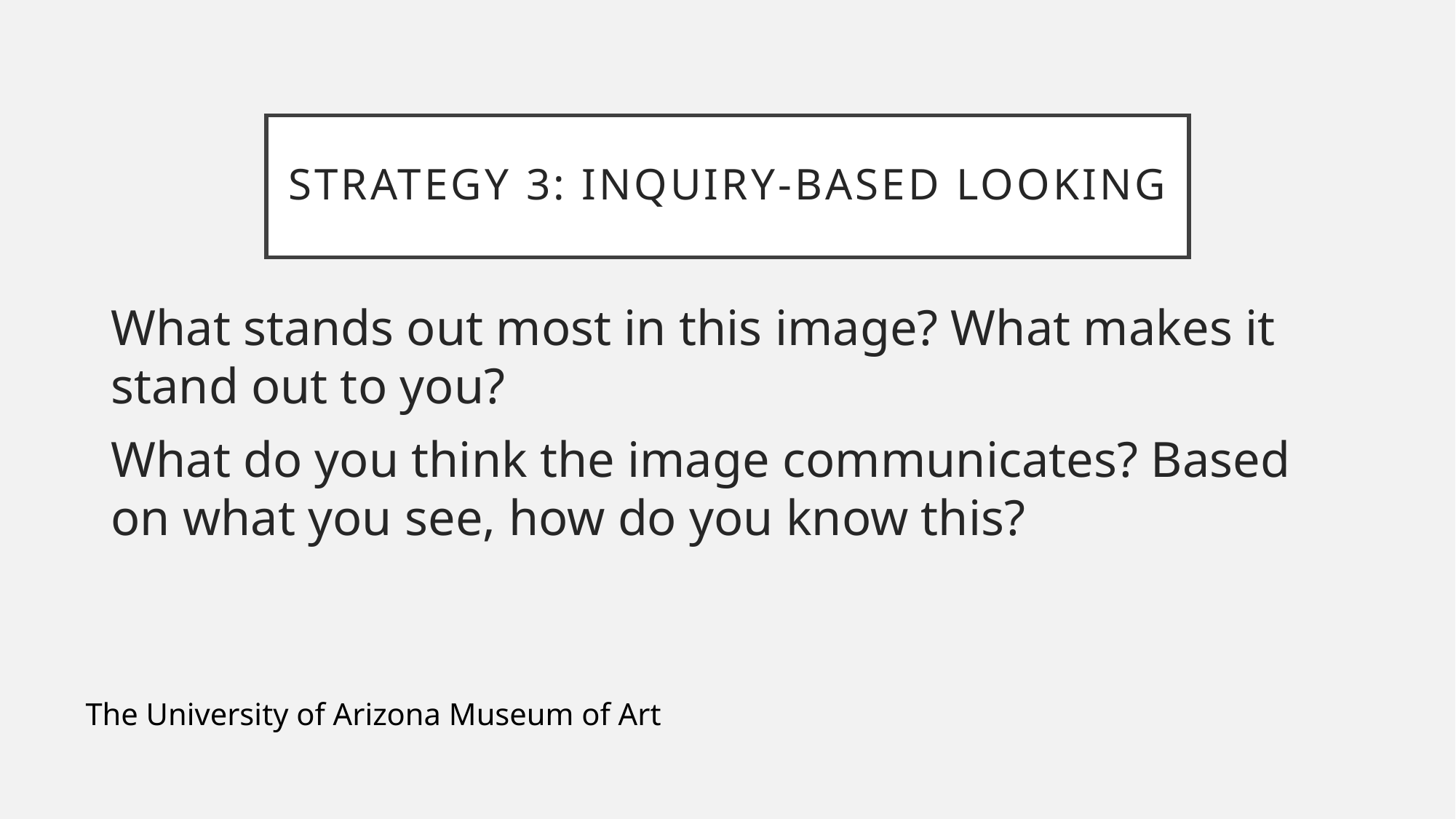

# Strategy 3: Inquiry-based Looking
What stands out most in this image? What makes it stand out to you?
What do you think the image communicates? Based on what you see, how do you know this?
The University of Arizona Museum of Art

## Slide 14
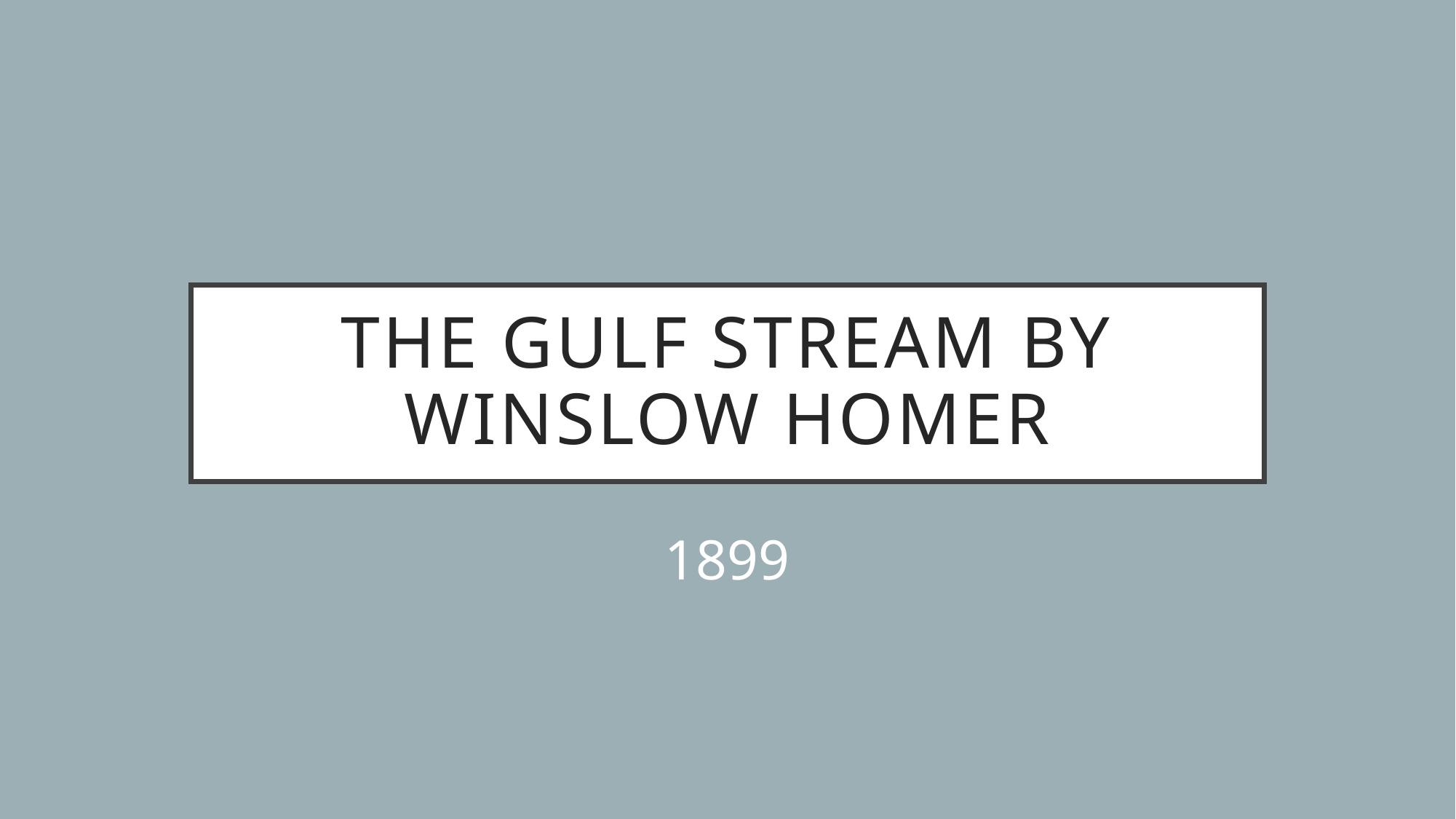

# The Gulf Stream by Winslow Homer
1899

## Slide 15
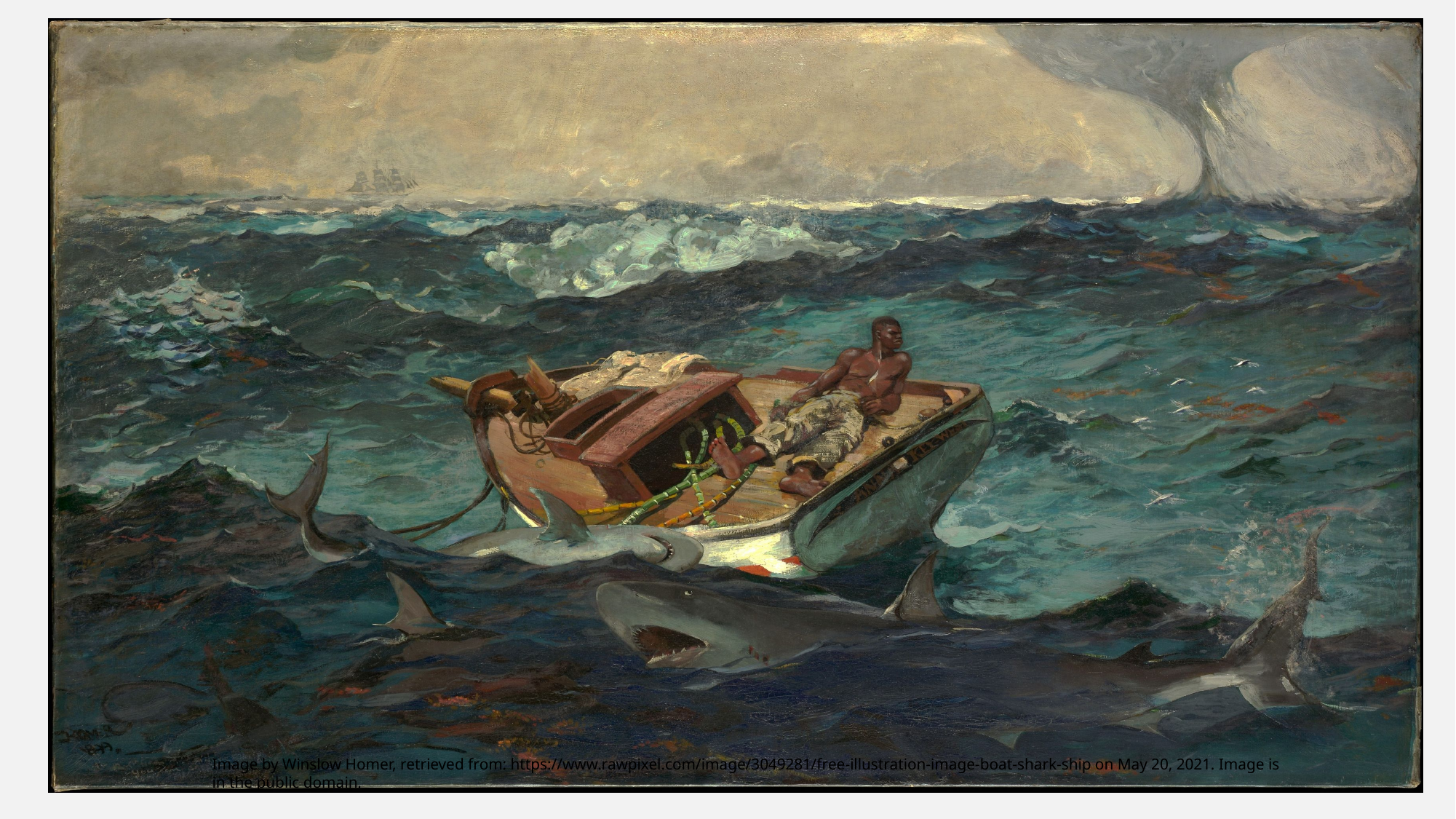

Image by Winslow Homer, retrieved from: https://www.rawpixel.com/image/3049281/free-illustration-image-boat-shark-ship on May 20, 2021. Image is in the public domain.

## Slide 16
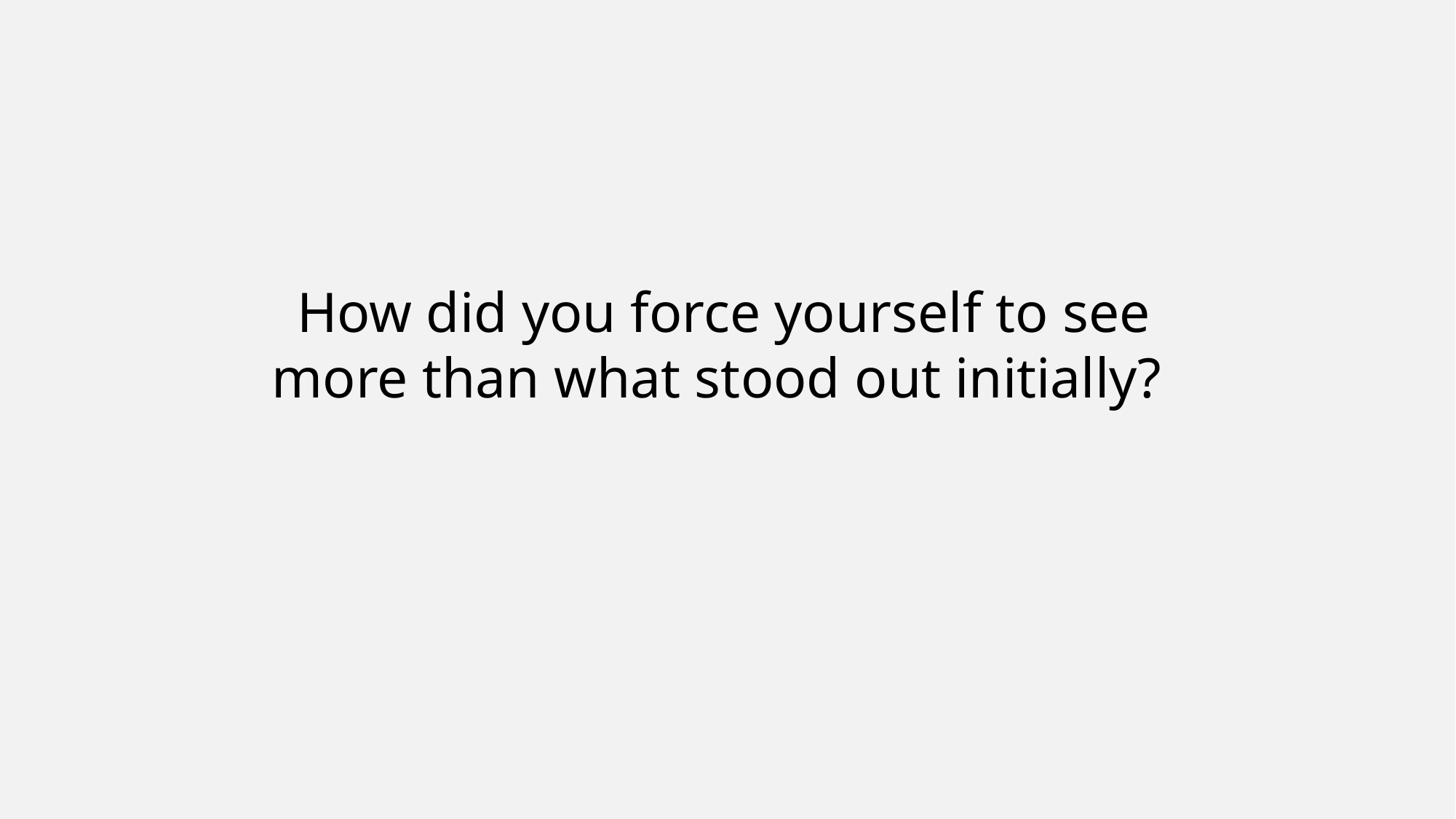

How did you force yourself to see more than what stood out initially?

## Slide 17
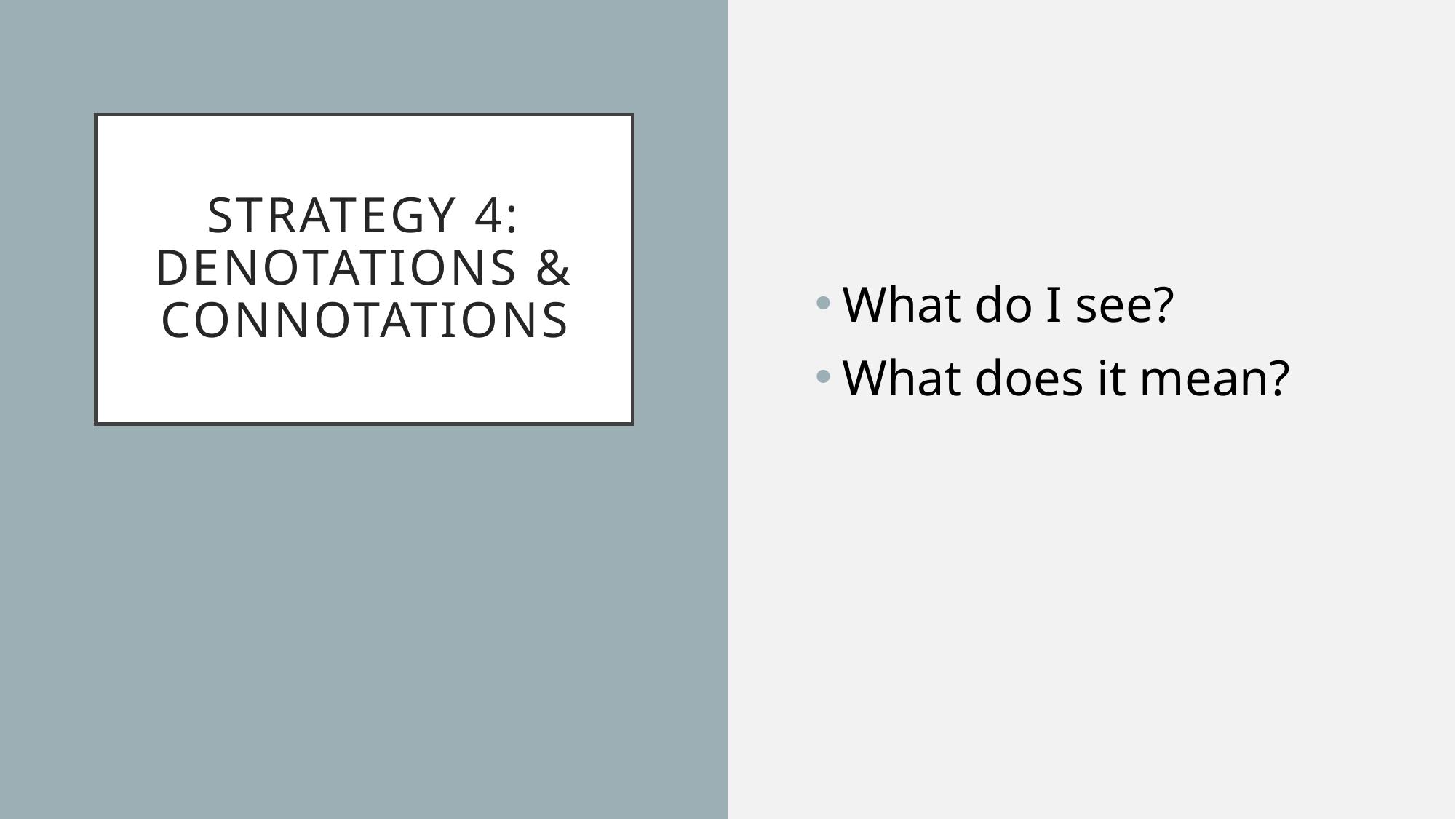

# Strategy 4: Denotations & connotations
What do I see?
What does it mean?

## Slide 18
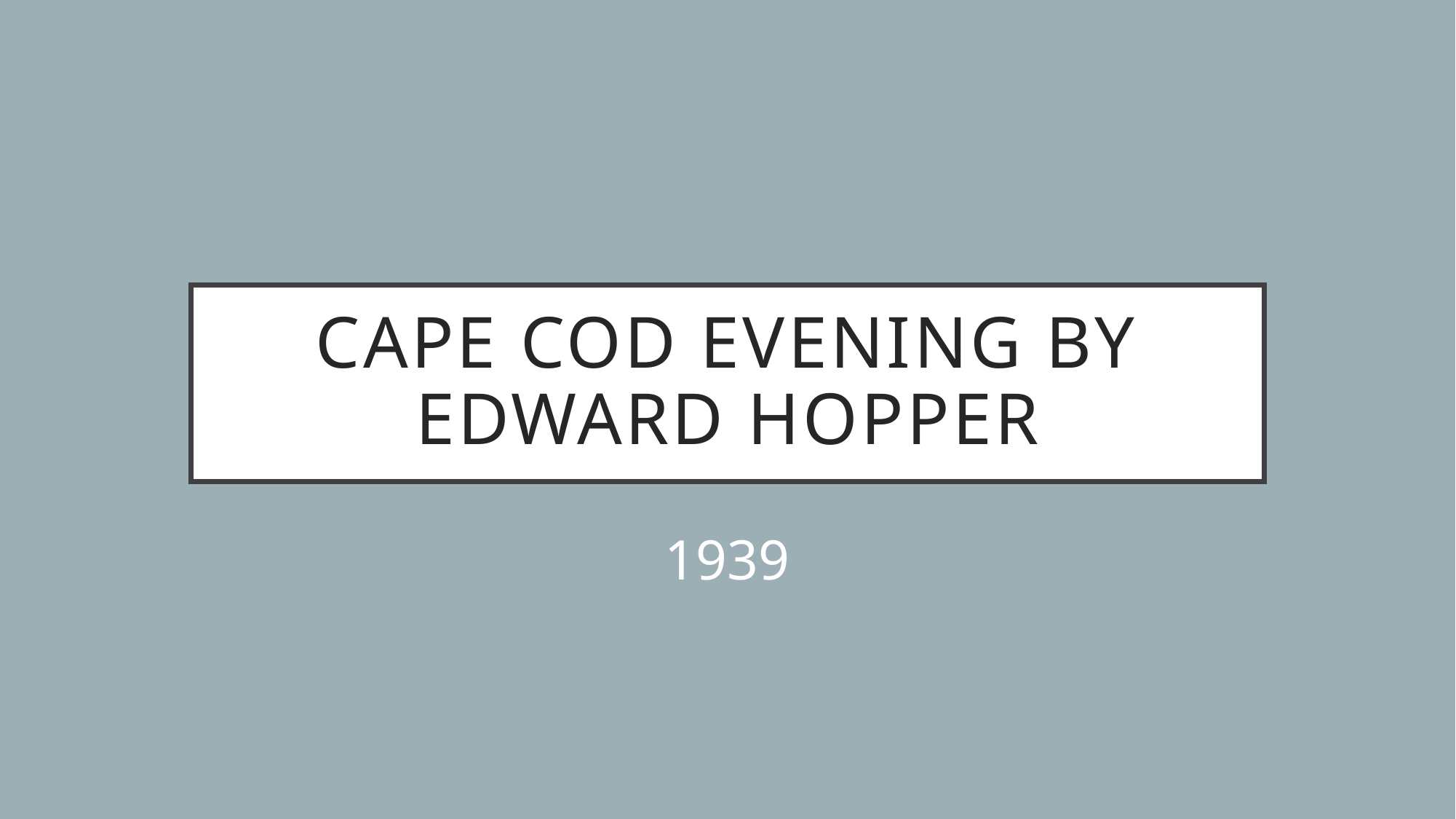

# Cape Cod Evening by Edward Hopper
1939

## Slide 19
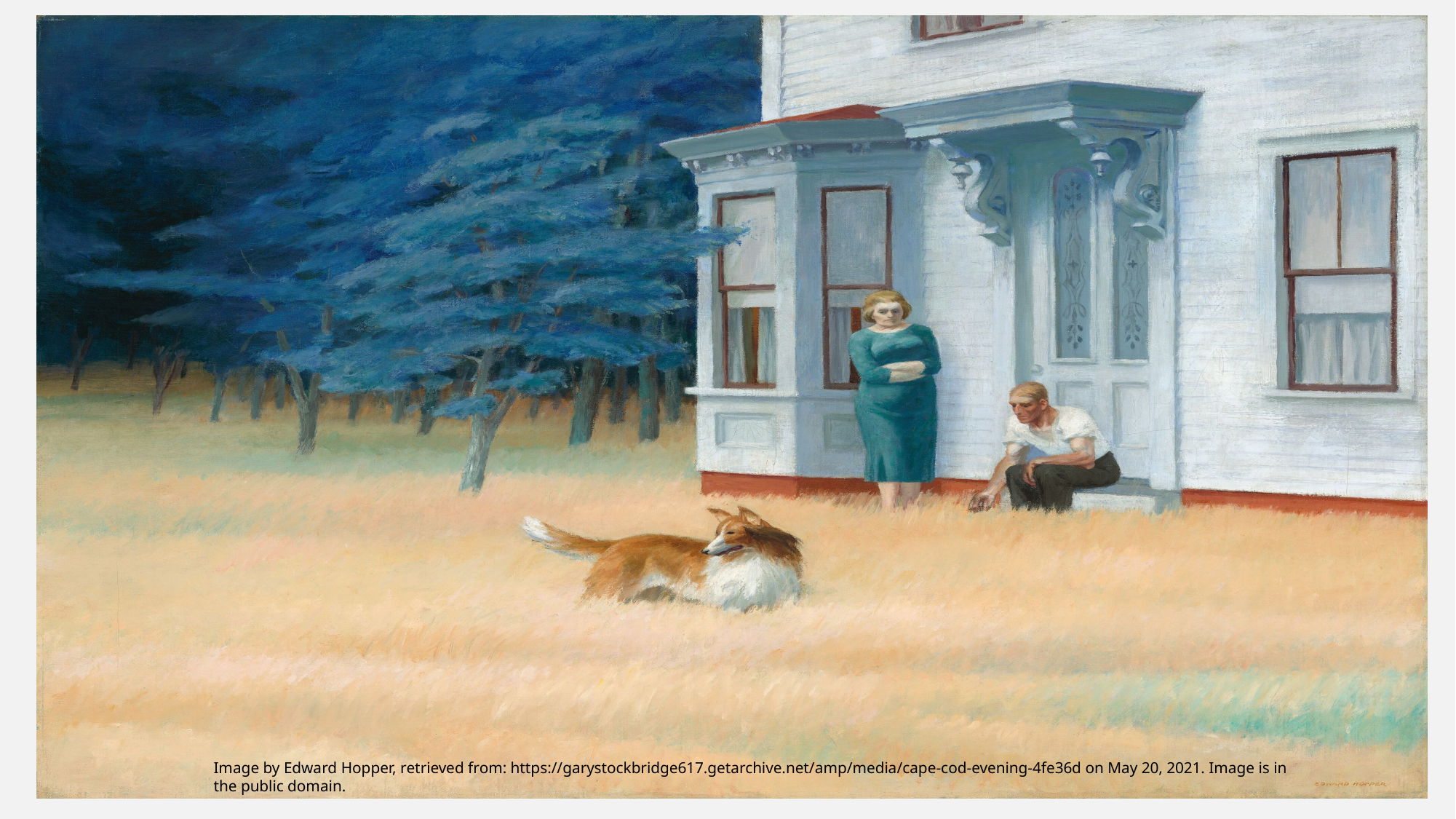

Image by Edward Hopper, retrieved from: https://garystockbridge617.getarchive.net/amp/media/cape-cod-evening-4fe36d on May 20, 2021. Image is in the public domain.

## Slide 20
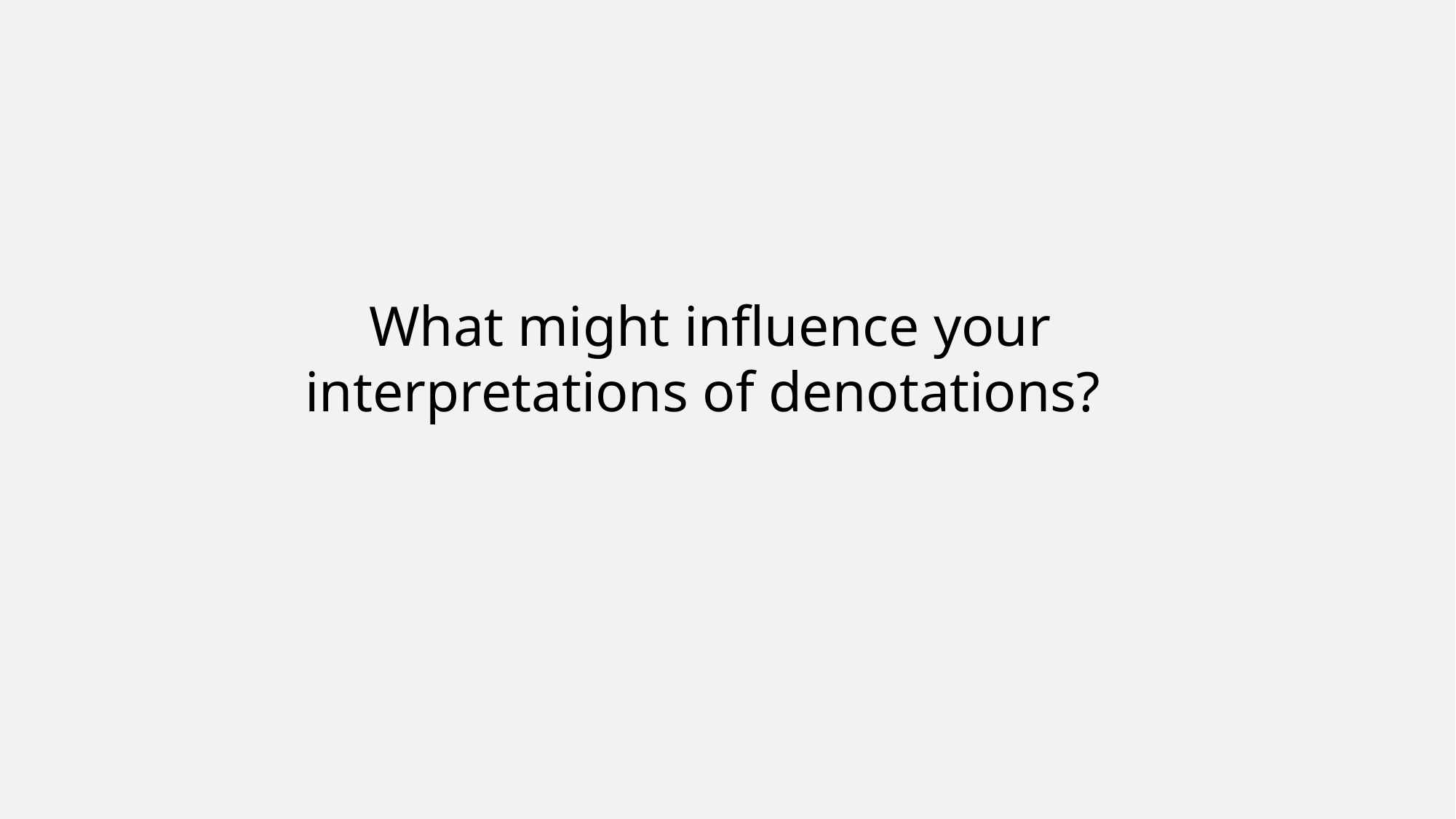

What might influence your interpretations of denotations?

## Slide 21
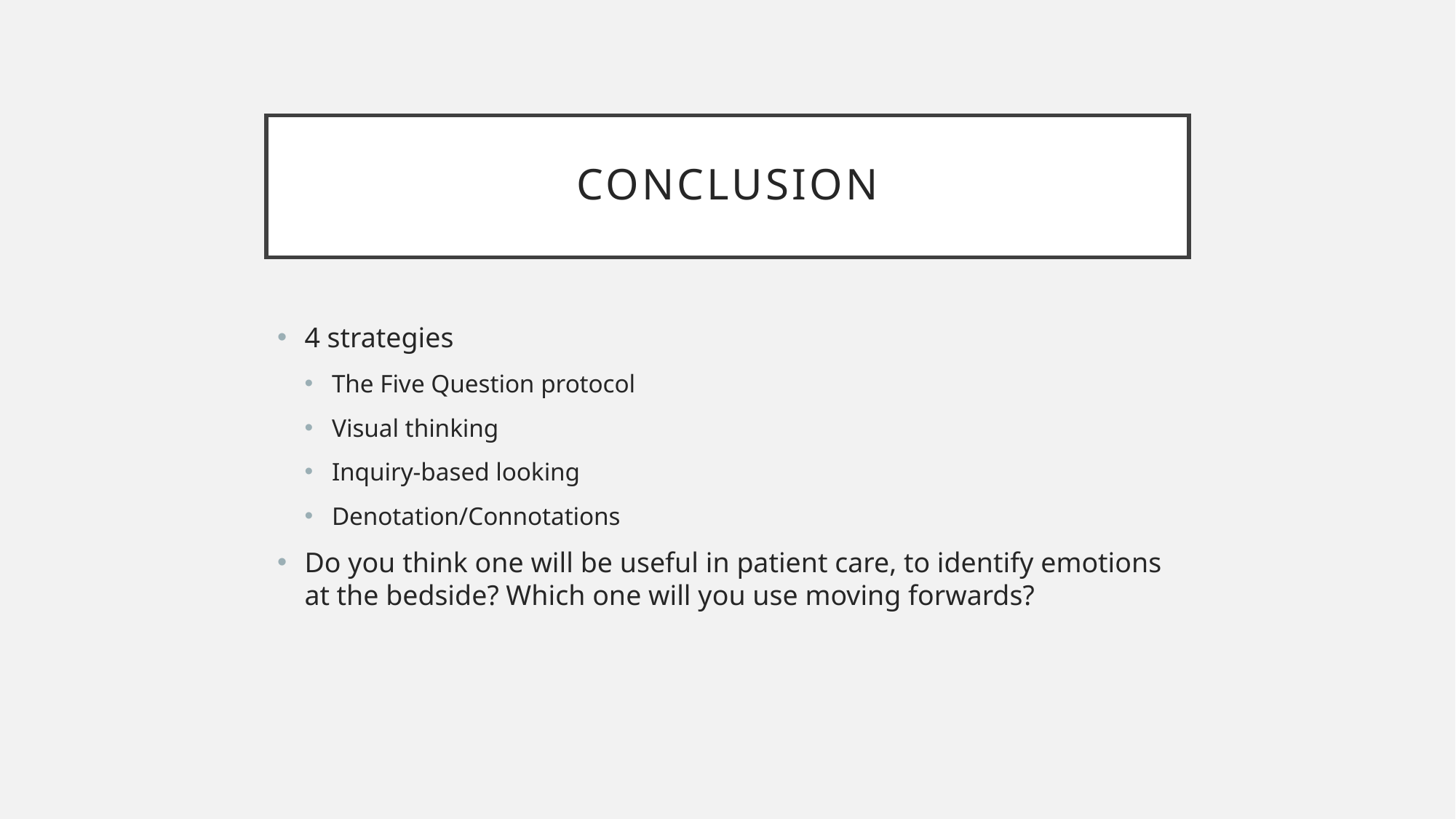

# CONCLUSIOn
4 strategies
The Five Question protocol
Visual thinking
Inquiry-based looking
Denotation/Connotations
Do you think one will be useful in patient care, to identify emotions at the bedside? Which one will you use moving forwards?

## Slide 22
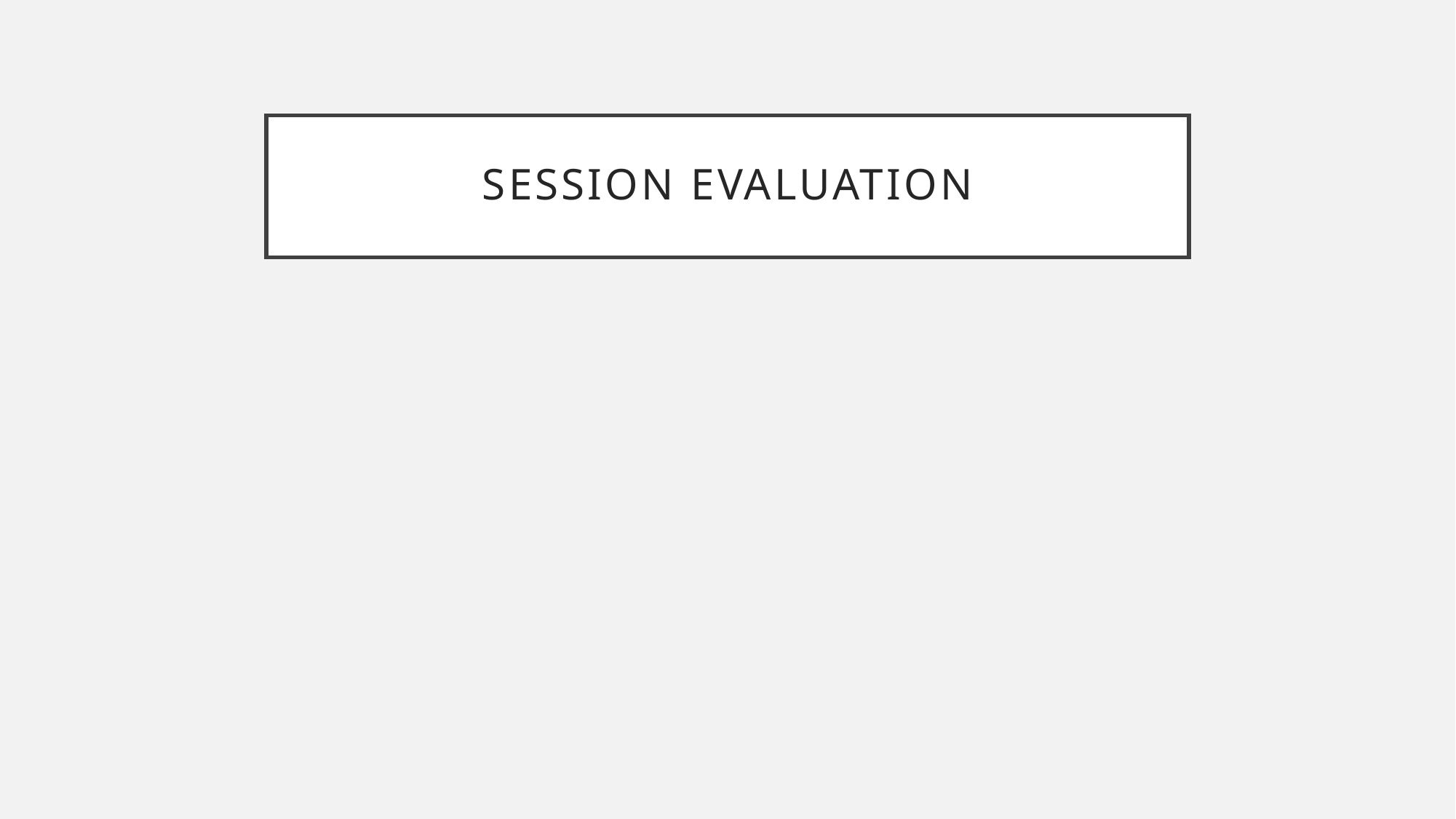

# Session Evaluation
