# Supplementary material for: An Empathy and Arts Curriculum During a Pediatrics Clerkship: Impact on Student Empathy and Behavior
Source: MedEdPORTAL. 2024 Jul 12;20:11414. doi: 10.15766/mep_2374-8265.11414 (PMC11239799; doi:10.15766/mep_2374-8265.11414)
Supplement: Supplementary file 1 — Empathy Session 1.pptxEmpathy Session 1 Facilitator Guide.docxEmpathy Session 2.pptxEmpathy Session 2 Facilitator Guide.docxEmpathy Video 1.mp4Empathy Video 2.mp4Empathy Video 3.mp4Empathy Session 2 Student Handout.docxEmpathy Session 1 Evaluation Form.docxEmpathy Session 2 Evaluation Form.docxToronto Empathy Questionnaire.docxEmpathy Behavior Checklists.docx [file mep_2374-8265.11414-s001.zip › C. Empathy Session 2.pptx]

## Slide 1
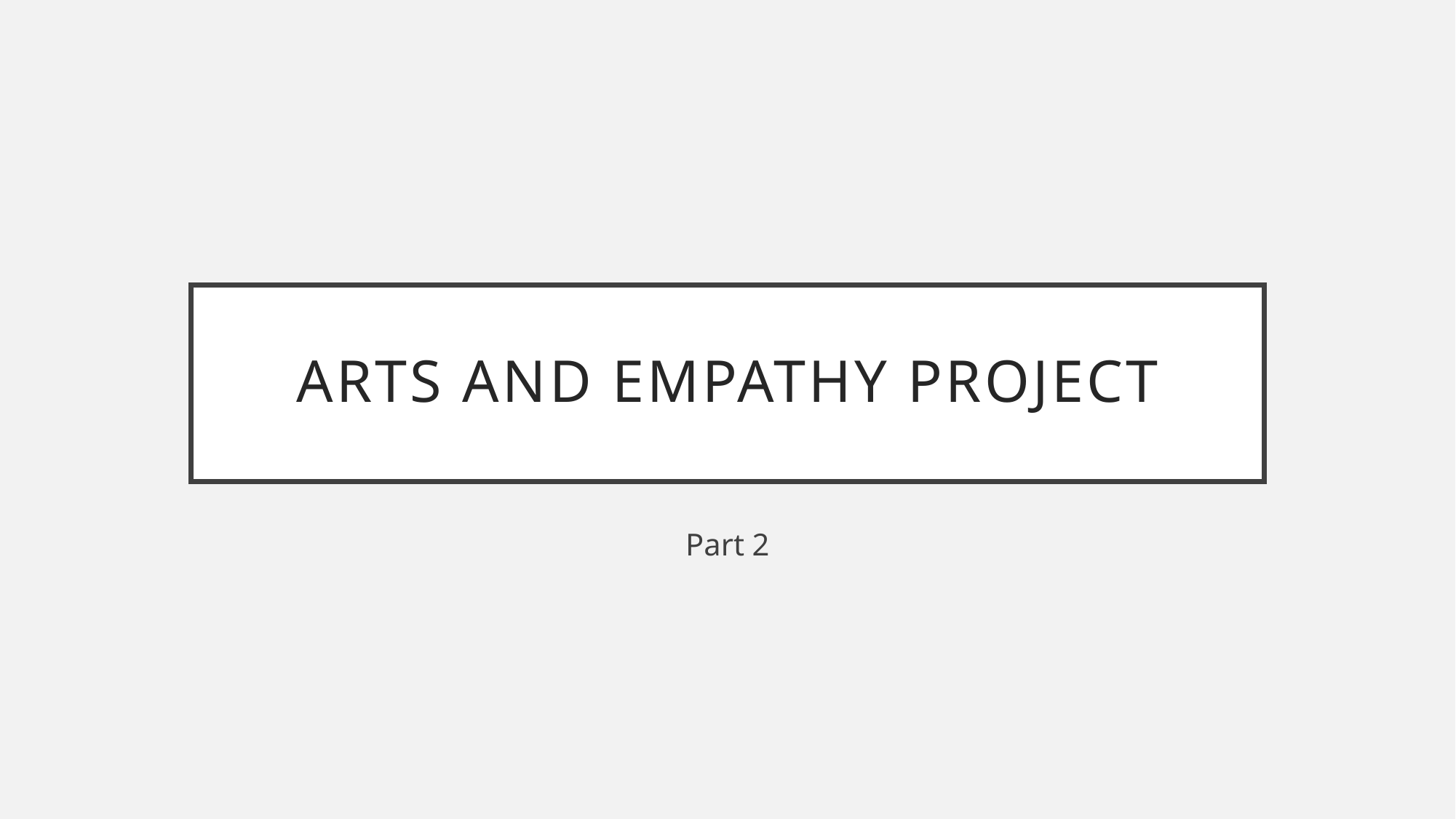

# Arts and Empathy Project
Part 2

## Slide 2
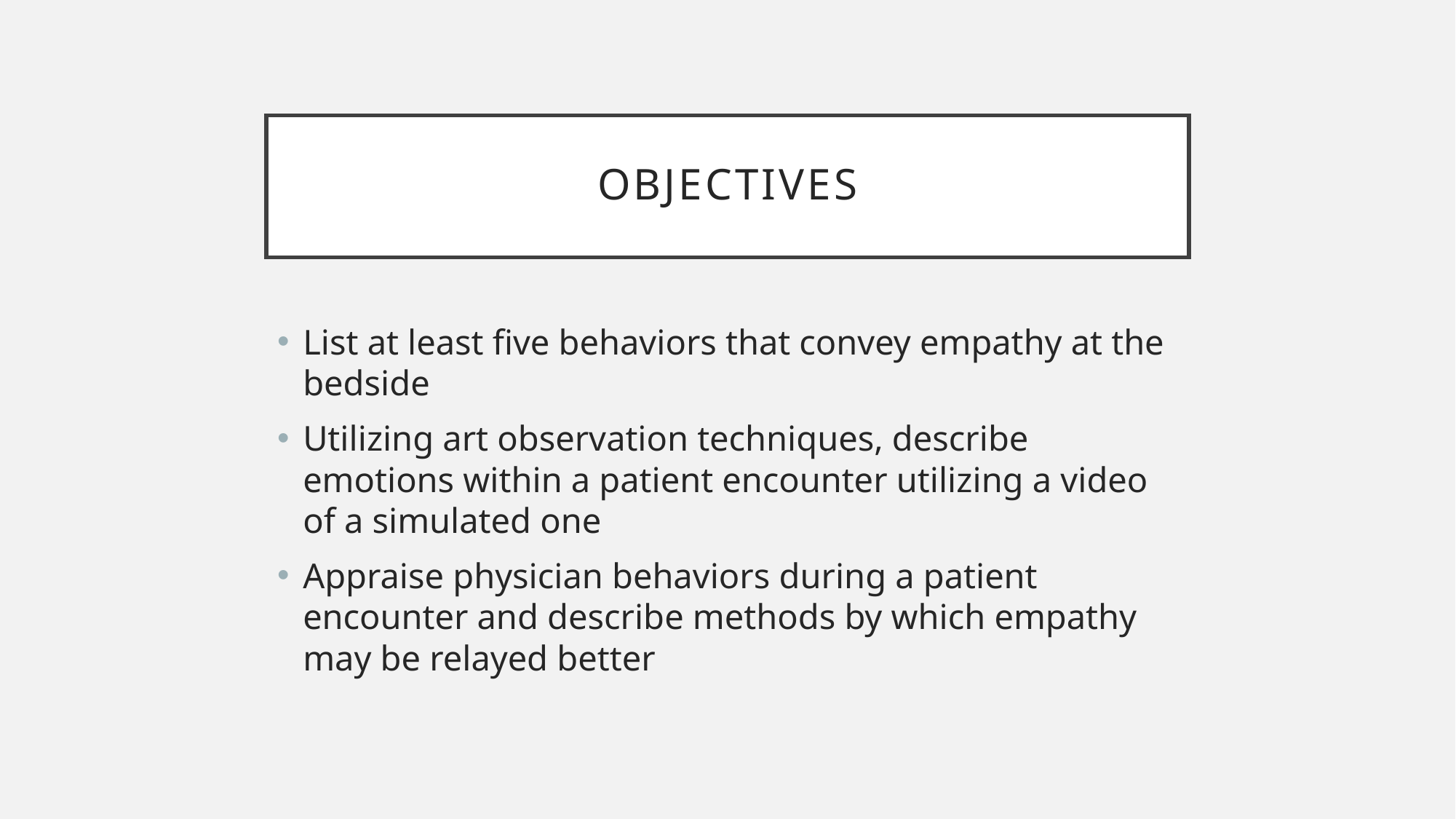

# Objectives
List at least five behaviors that convey empathy at the bedside
Utilizing art observation techniques, describe emotions within a patient encounter utilizing a video of a simulated one
Appraise physician behaviors during a patient encounter and describe methods by which empathy may be relayed better

## Slide 3
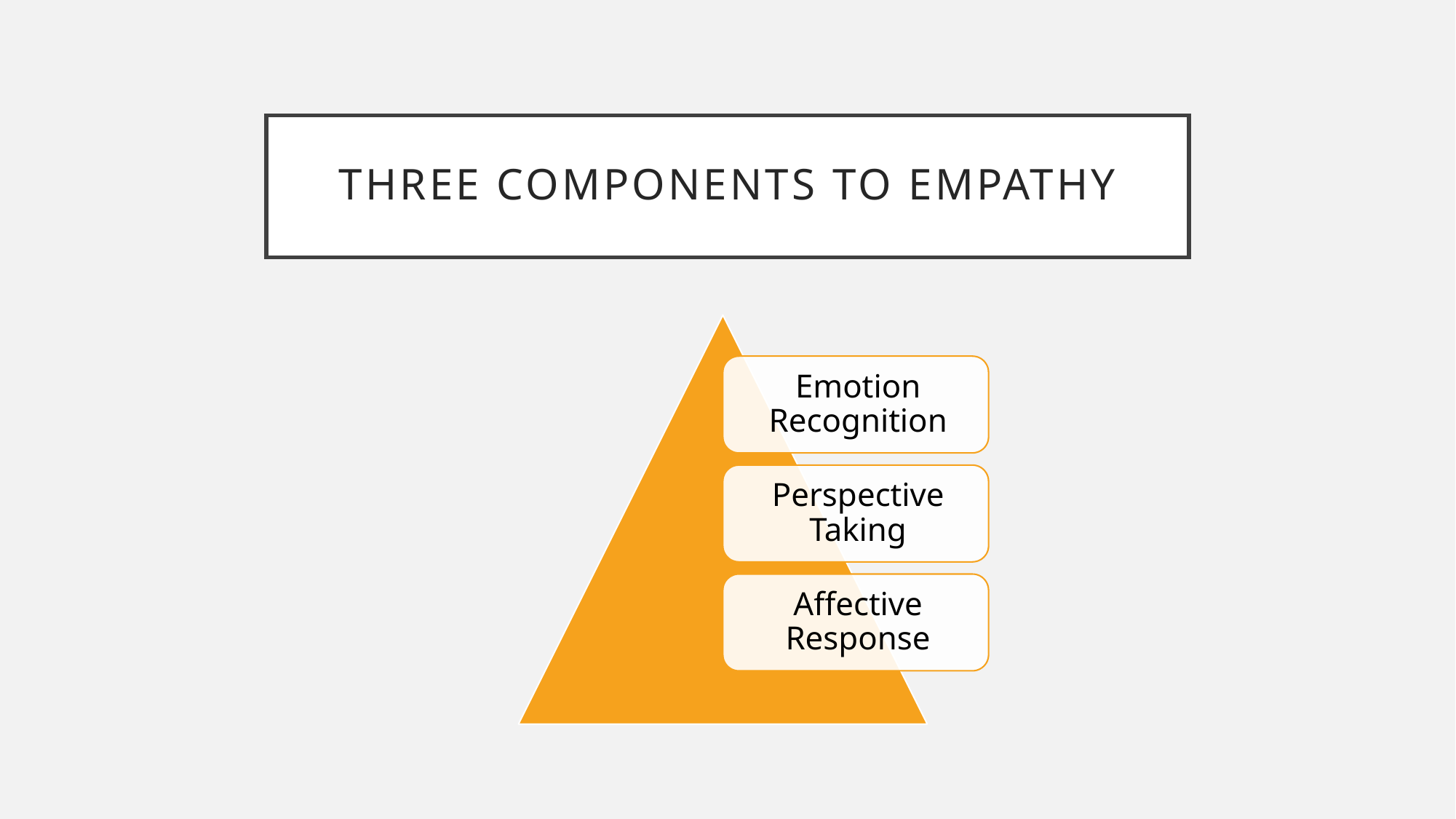

# Three components to empathy

## Slide 4
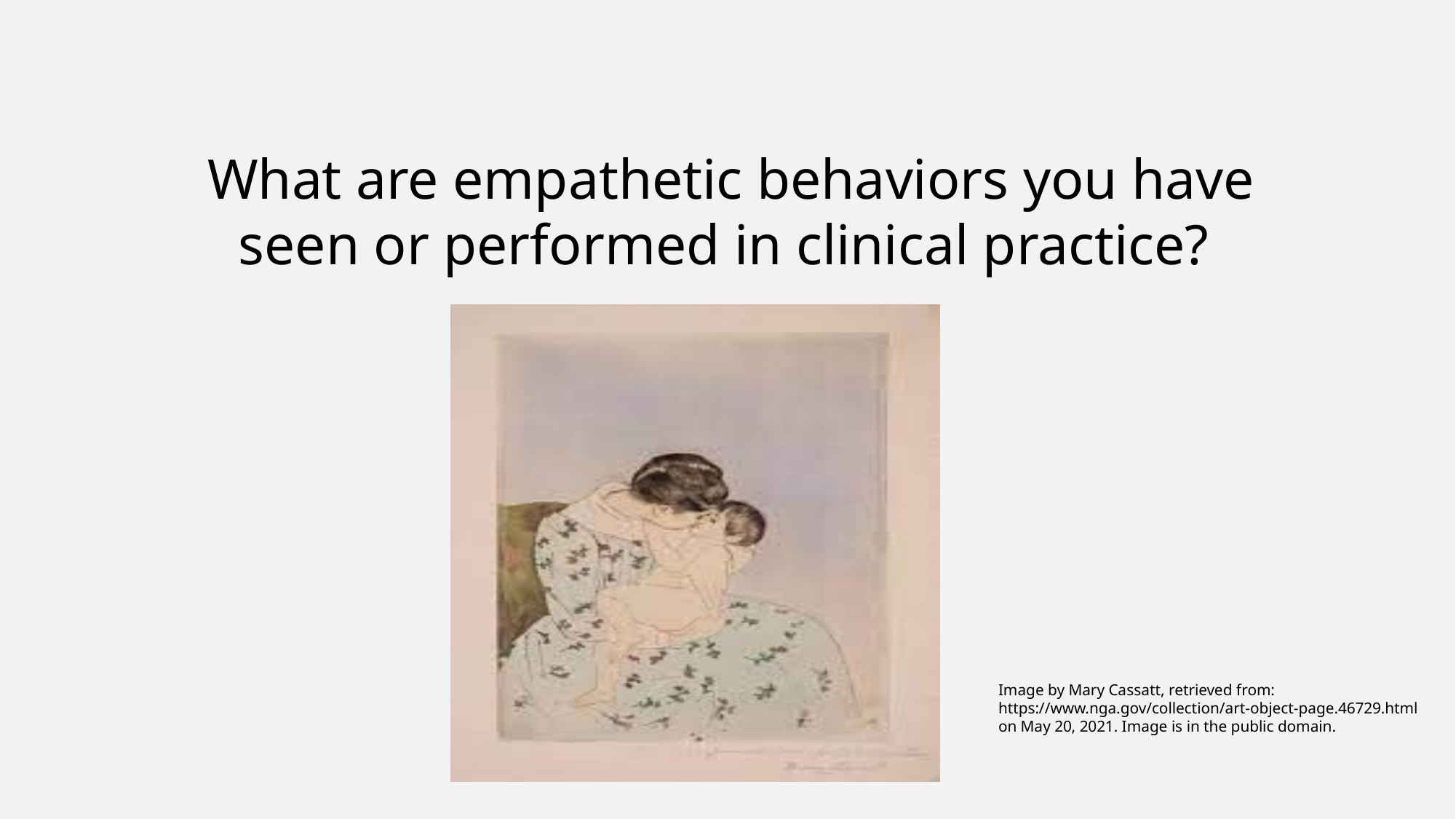

What are empathetic behaviors you have seen or performed in clinical practice?
Image by Mary Cassatt, retrieved from: https://www.nga.gov/collection/art-object-page.46729.html
on May 20, 2021. Image is in the public domain.

## Slide 5
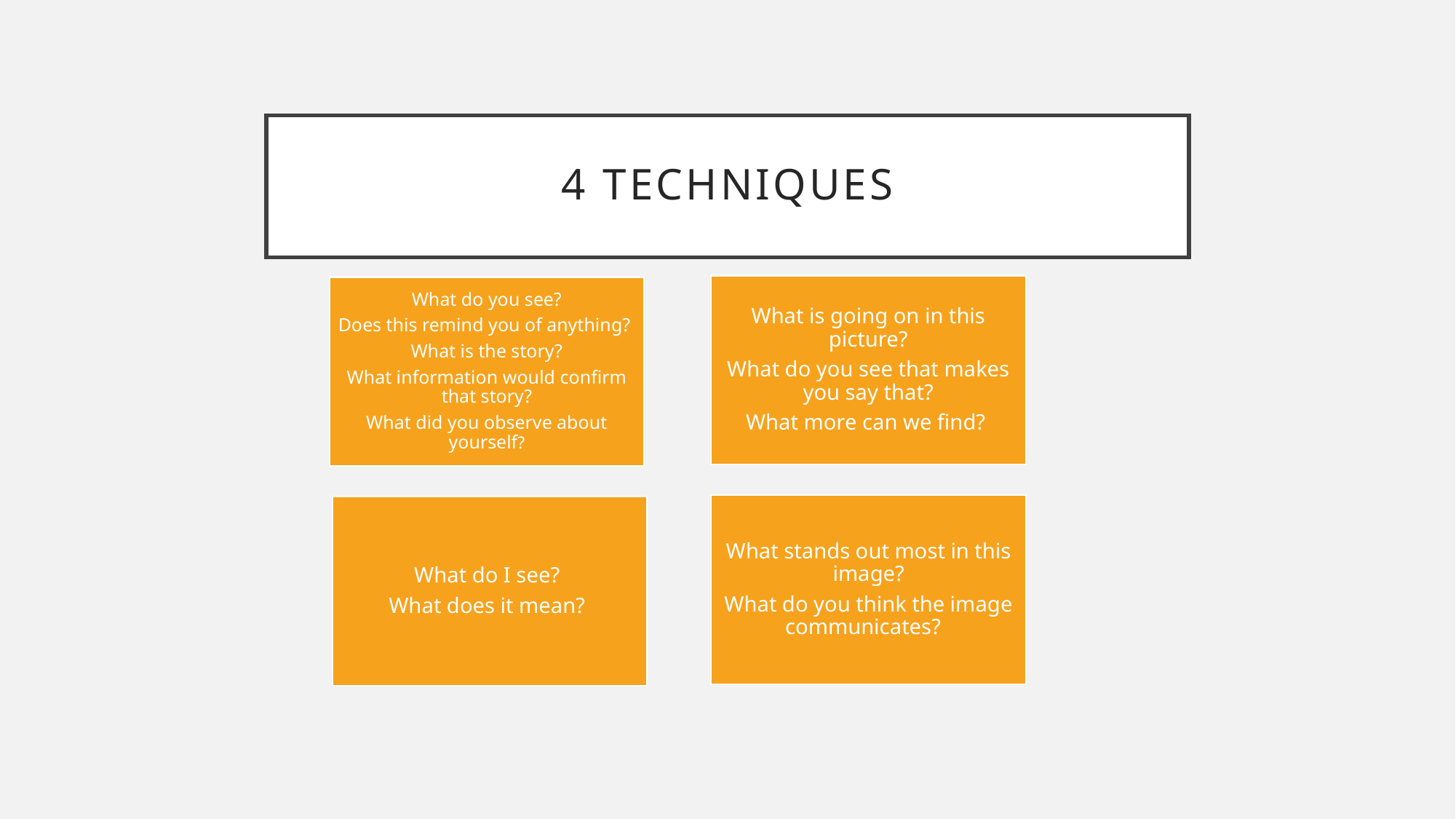

# 4 techniques

## Slide 6
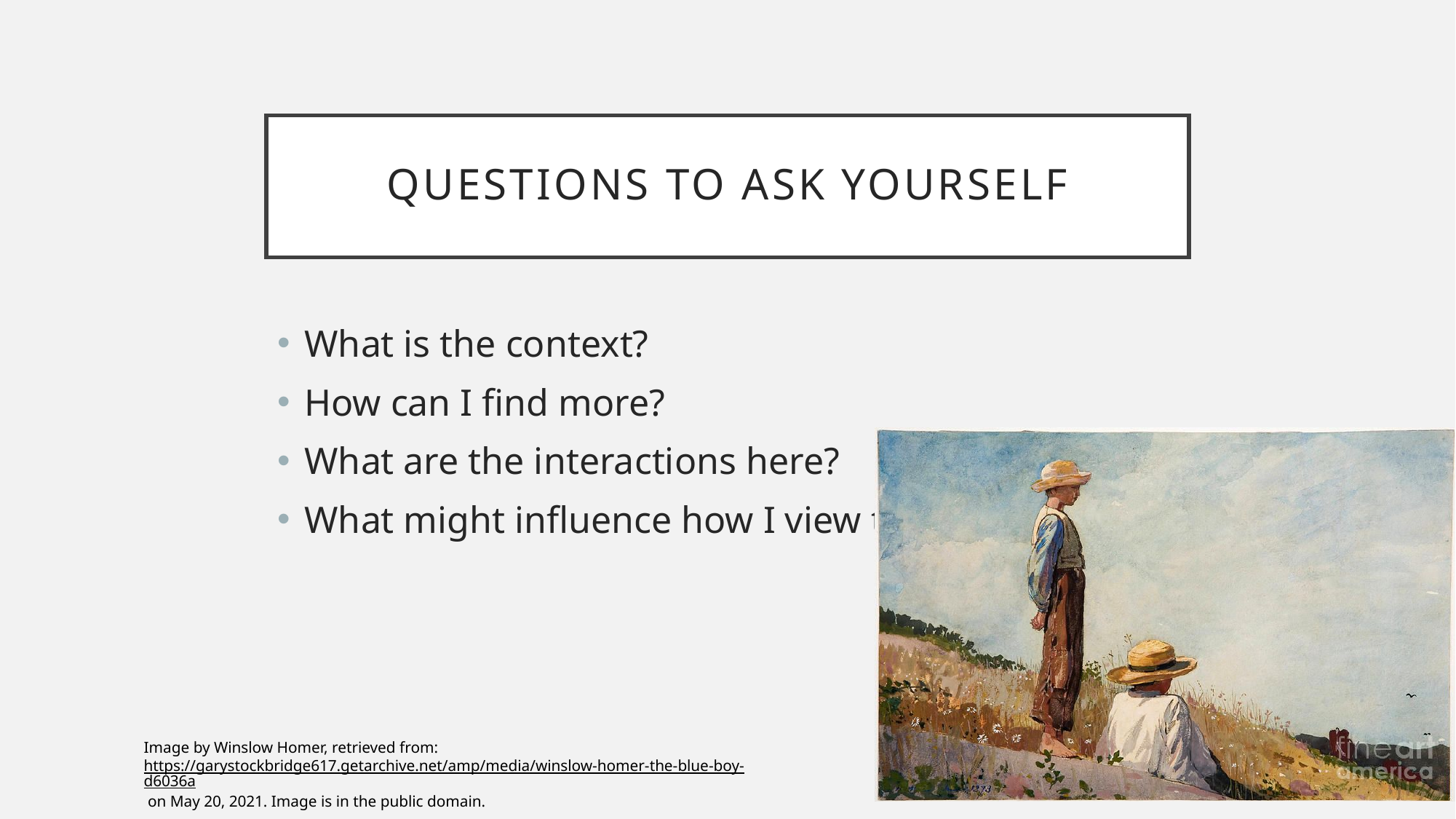

# Questions to ask yourself
What is the context?
How can I find more?
What are the interactions here?
What might influence how I view this?
Image by Winslow Homer, retrieved from: https://garystockbridge617.getarchive.net/amp/media/winslow-homer-the-blue-boy-d6036a on May 20, 2021. Image is in the public domain.

## Slide 7
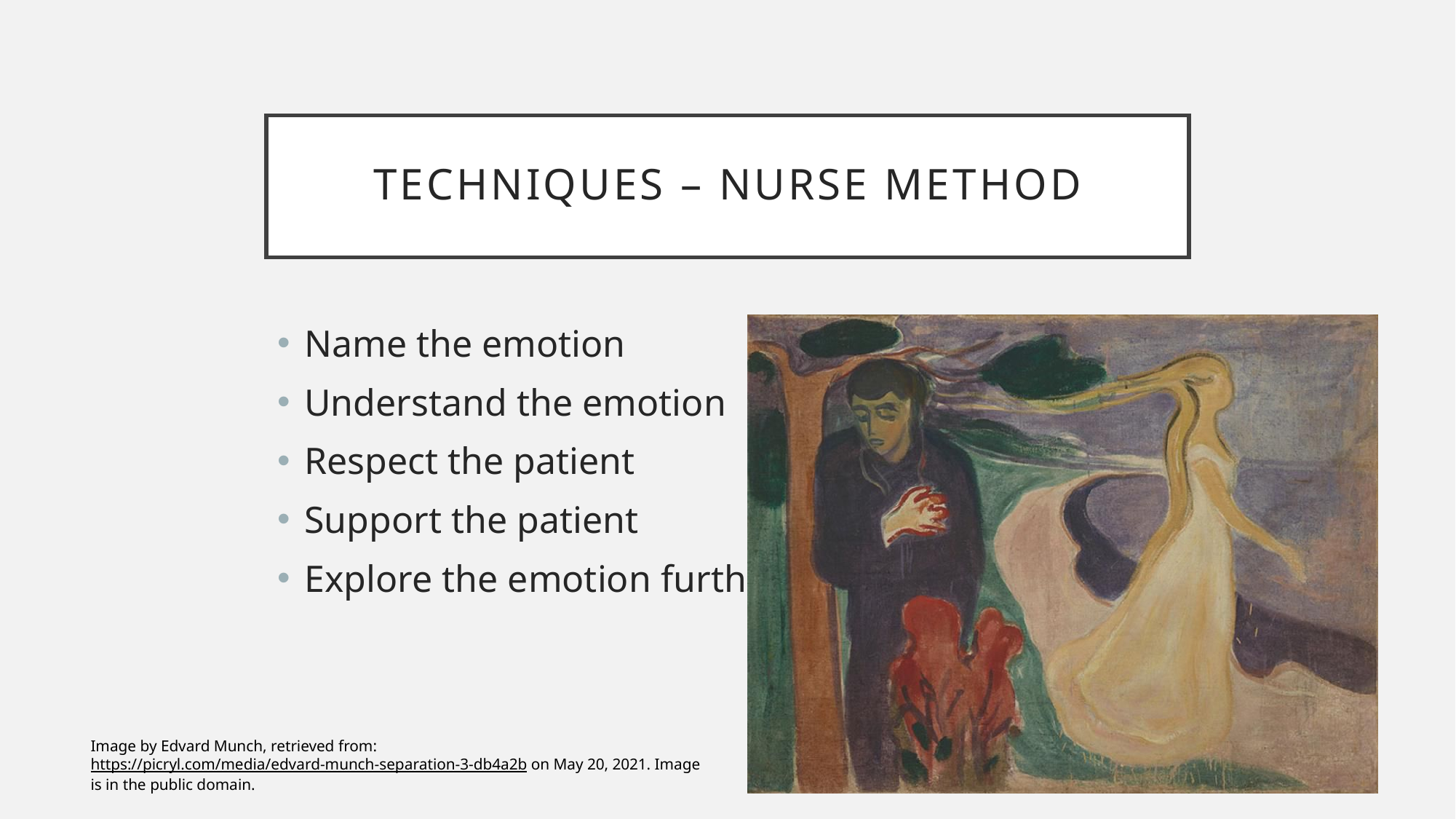

# Techniques – NURSE method
Name the emotion
Understand the emotion
Respect the patient
Support the patient
Explore the emotion further
Image by Edvard Munch, retrieved from: https://picryl.com/media/edvard-munch-separation-3-db4a2b on May 20, 2021. Image is in the public domain.

## Slide 8
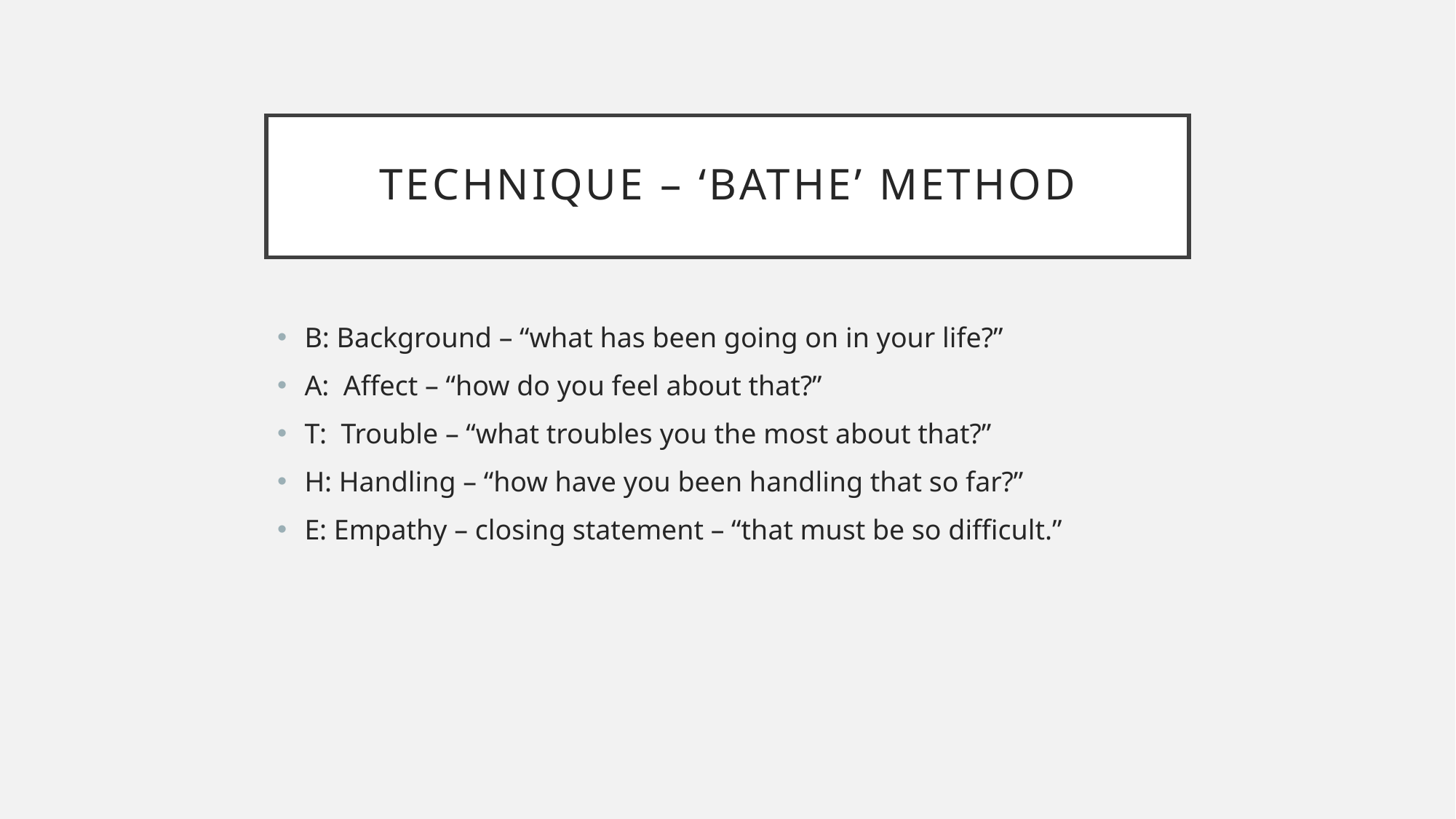

# Technique – ‘bathe’ method
B: Background – “what has been going on in your life?”
A: Affect – “how do you feel about that?”
T: Trouble – “what troubles you the most about that?”
H: Handling – “how have you been handling that so far?”
E: Empathy – closing statement – “that must be so difficult.”

## Slide 9
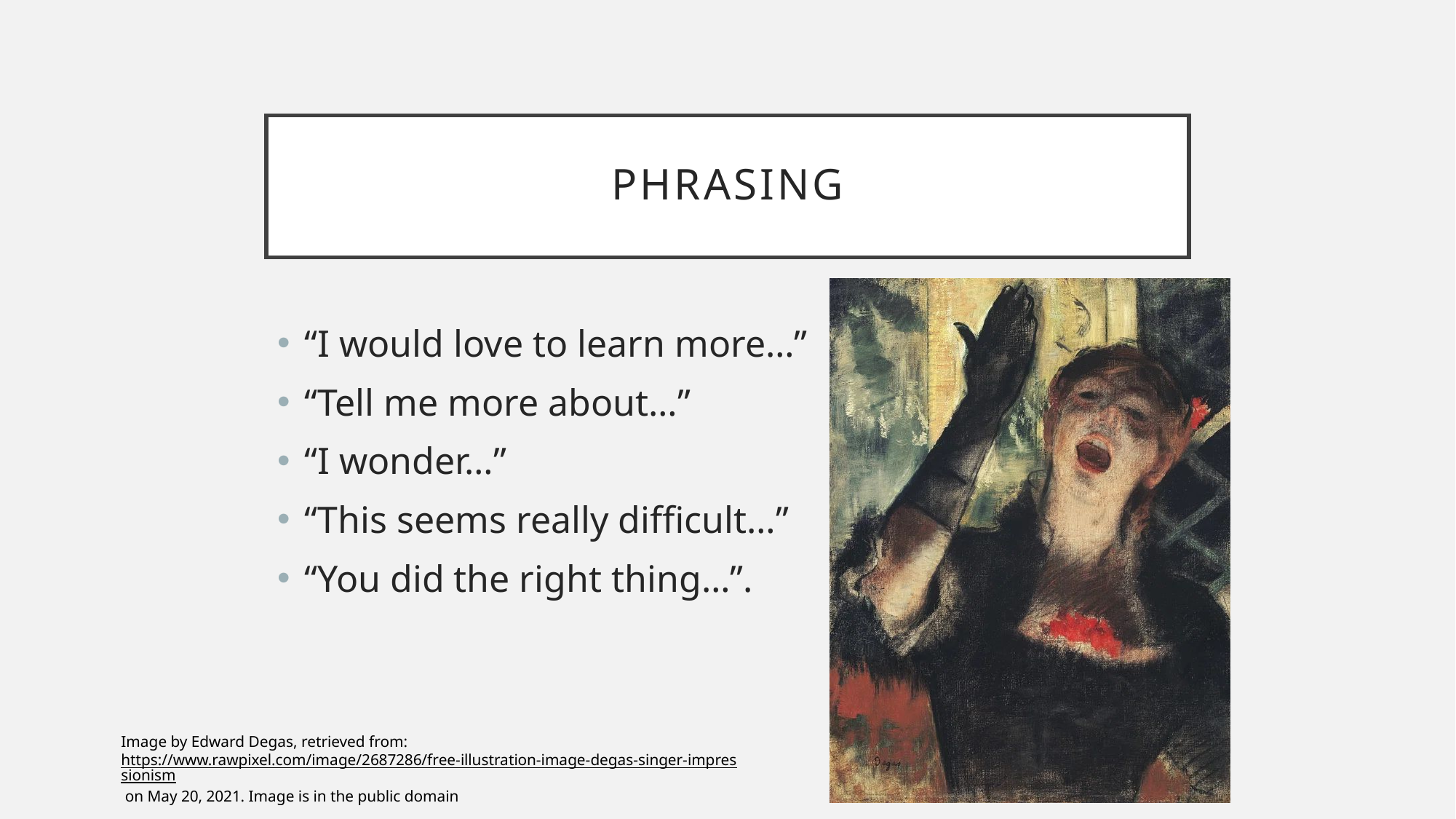

# Phrasing
“I would love to learn more…”
“Tell me more about…”
“I wonder…”
“This seems really difficult…”
“You did the right thing…”.
Image by Edward Degas, retrieved from: https://www.rawpixel.com/image/2687286/free-illustration-image-degas-singer-impressionism on May 20, 2021. Image is in the public domain

## Slide 10
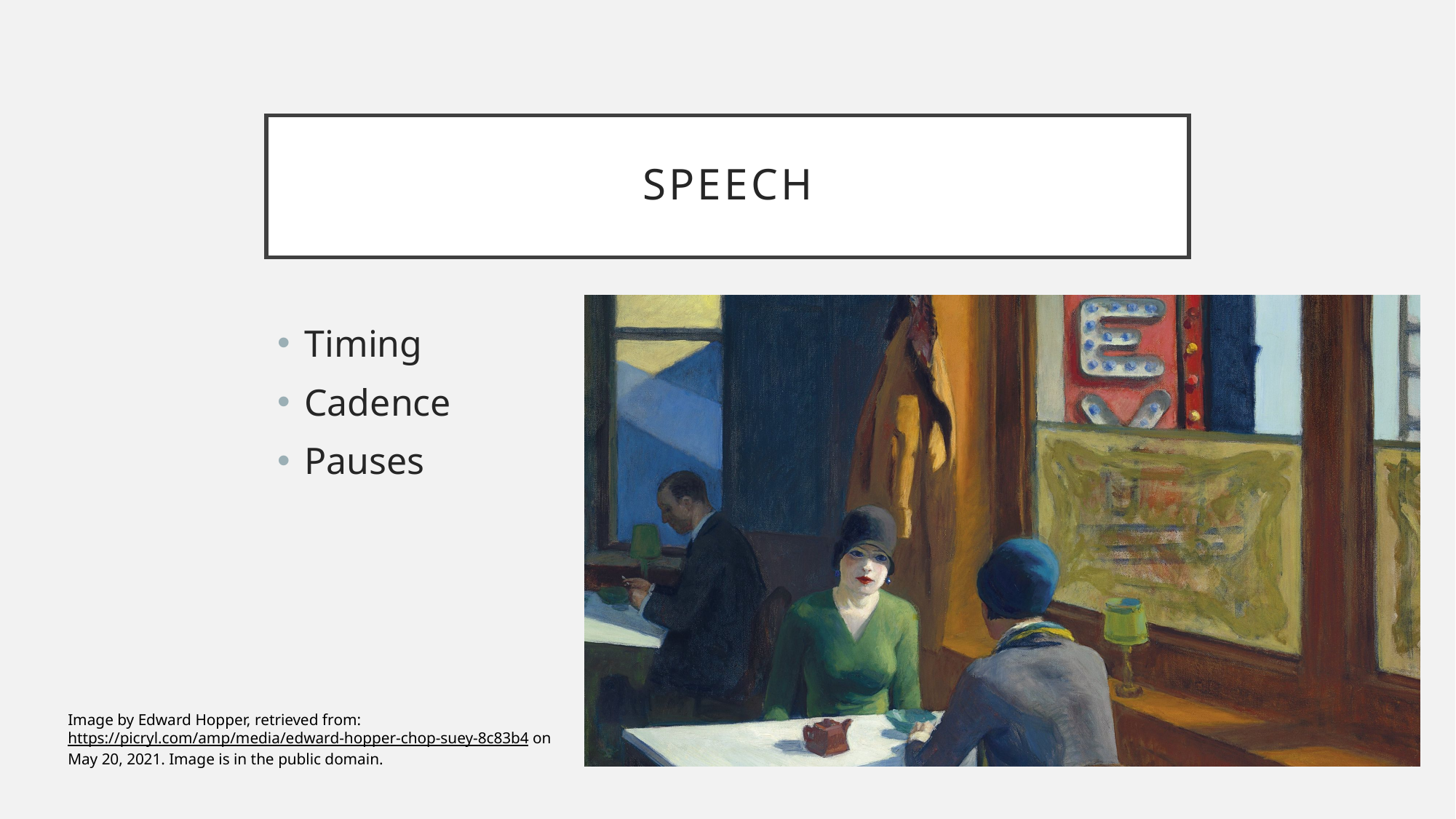

# Speech
Timing
Cadence
Pauses
Image by Edward Hopper, retrieved from: https://picryl.com/amp/media/edward-hopper-chop-suey-8c83b4 on May 20, 2021. Image is in the public domain.

## Slide 11
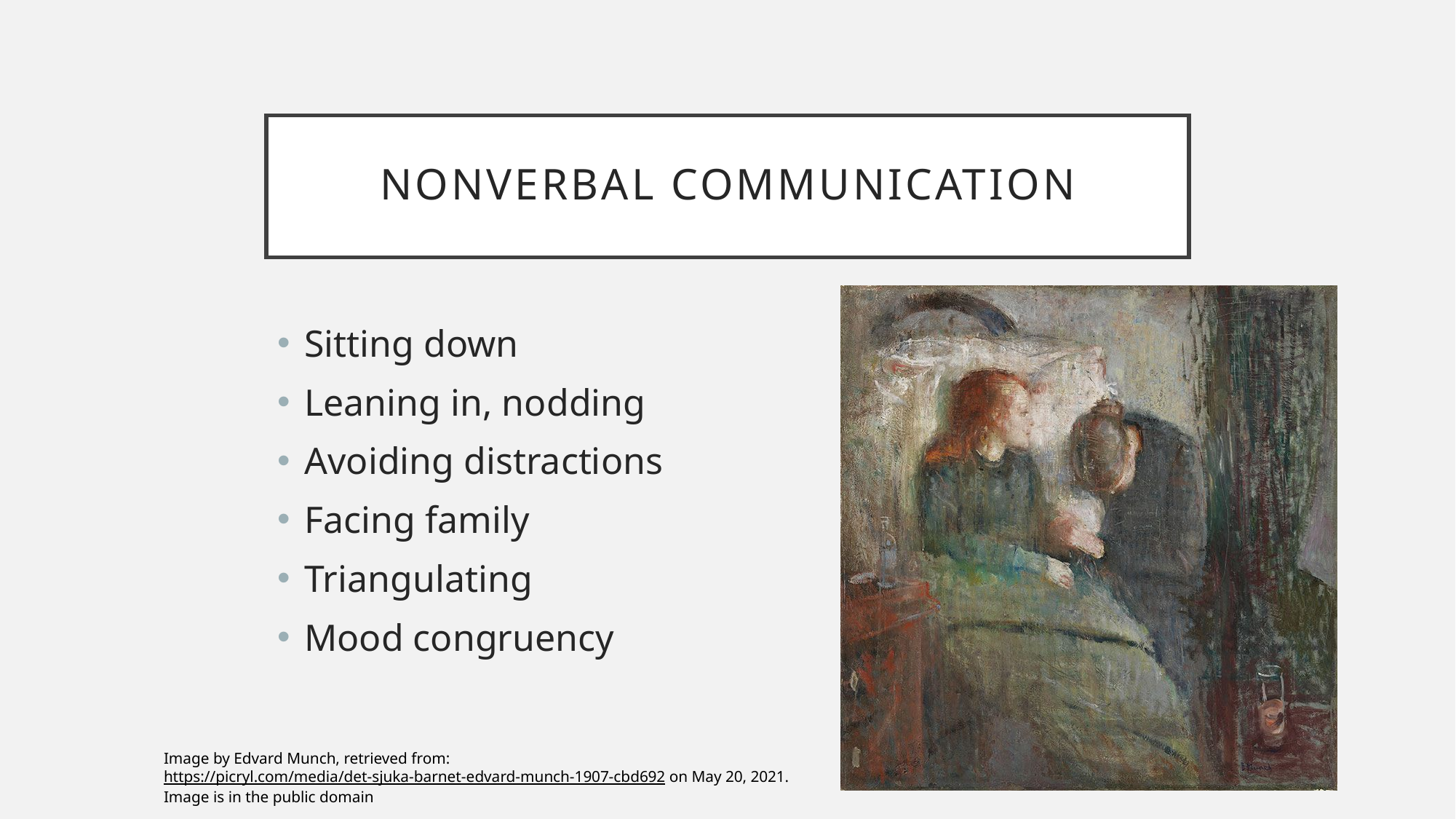

# Nonverbal communication
Sitting down
Leaning in, nodding
Avoiding distractions
Facing family
Triangulating
Mood congruency
Image by Edvard Munch, retrieved from: https://picryl.com/media/det-sjuka-barnet-edvard-munch-1907-cbd692 on May 20, 2021. Image is in the public domain

## Slide 12
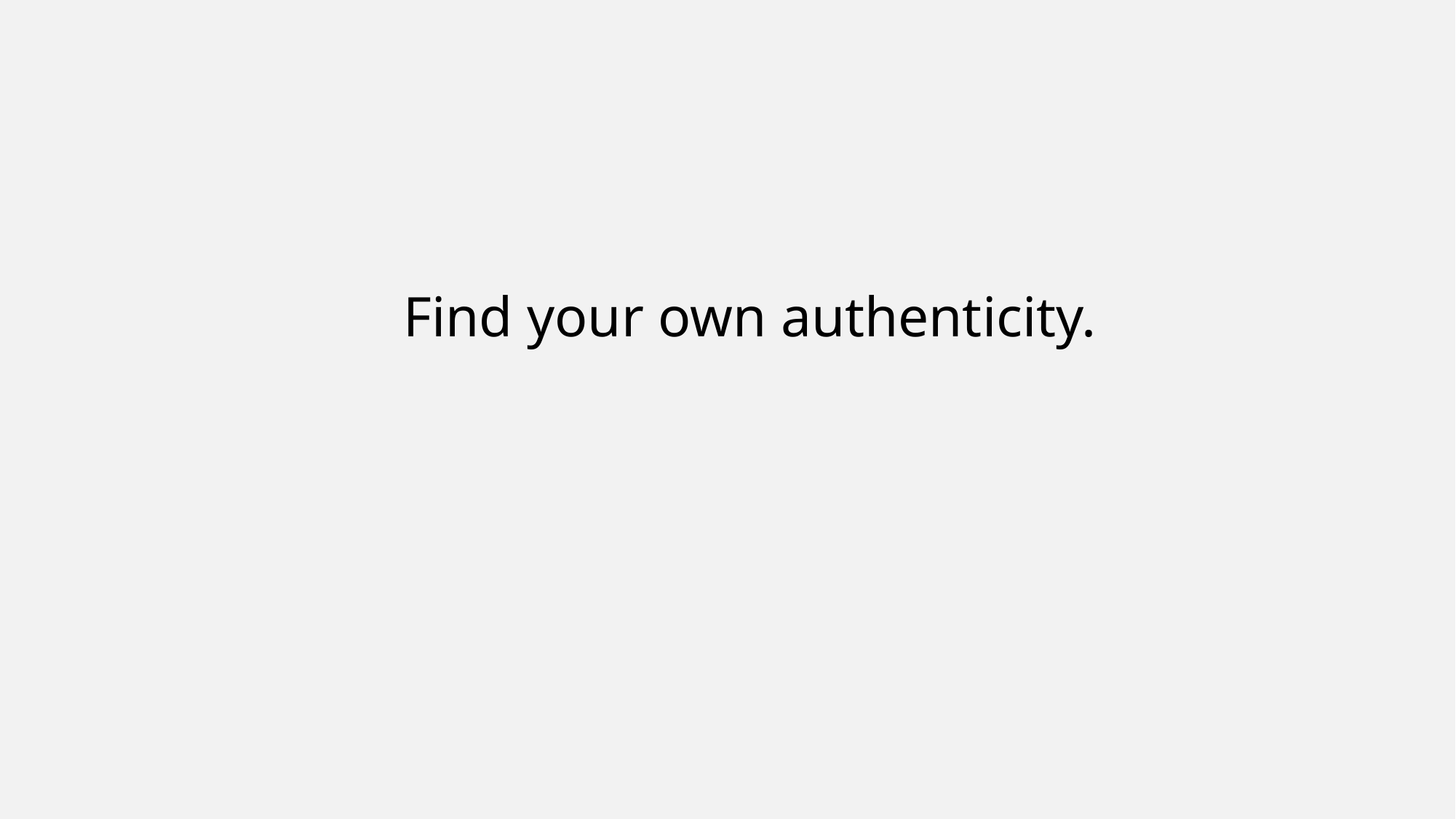

Find your own authenticity.

## Slide 13
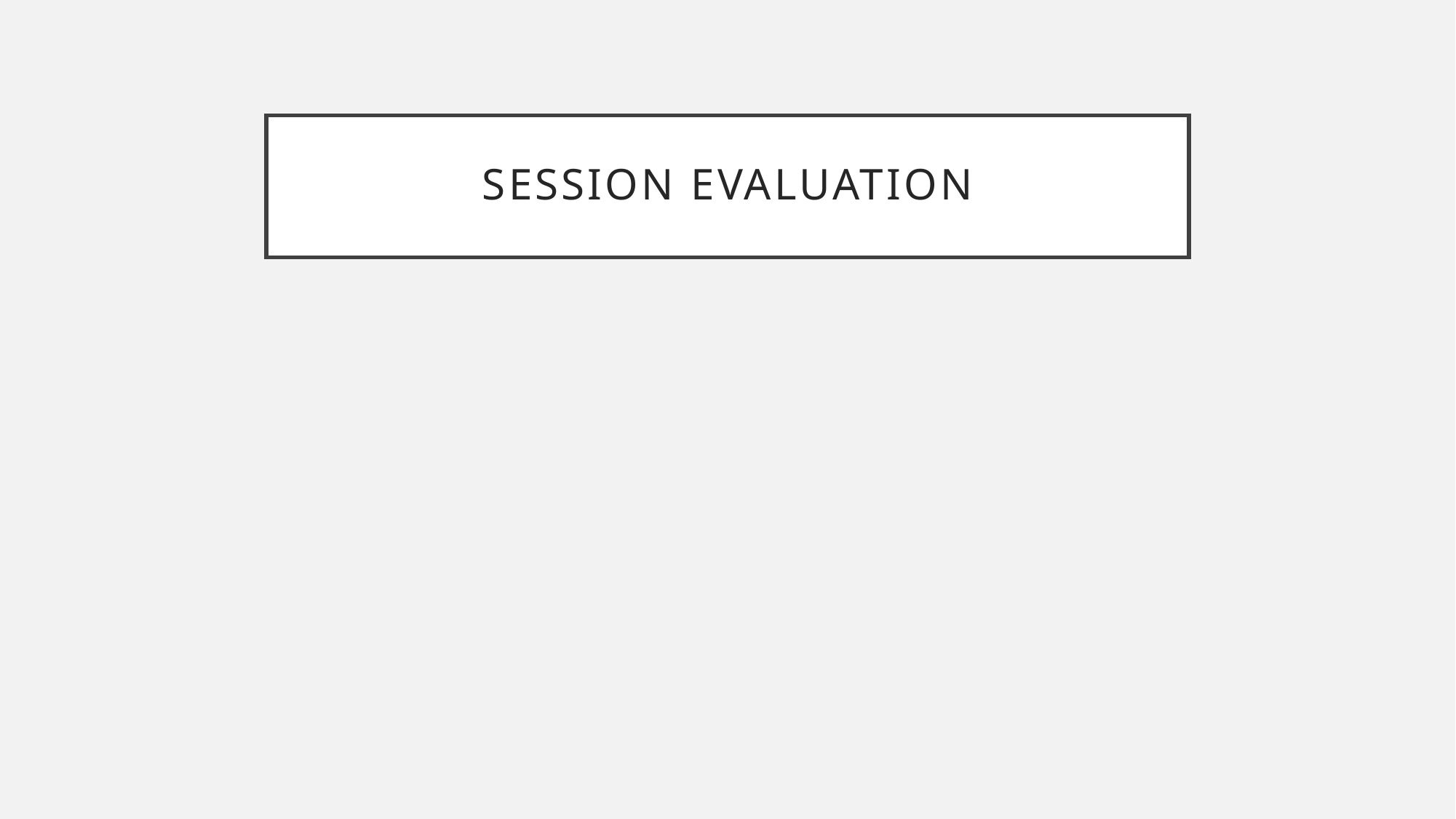

# Session Evaluation
