# Supplementary material for: An Empathy and Arts Curriculum During a Pediatrics Clerkship: Impact on Student Empathy and Behavior
Source: MedEdPORTAL. 2024 Jul 12;20:11414. doi: 10.15766/mep_2374-8265.11414 (PMC11239799; doi:10.15766/mep_2374-8265.11414)
Supplement: Supplementary file 1 — Empathy Session 1.pptxEmpathy Session 1 Facilitator Guide.docxEmpathy Session 2.pptxEmpathy Session 2 Facilitator Guide.docxEmpathy Video 1.mp4Empathy Video 2.mp4Empathy Video 3.mp4Empathy Session 2 Student Handout.docxEmpathy Session 1 Evaluation Form.docxEmpathy Session 2 Evaluation Form.docxToronto Empathy Questionnaire.docxEmpathy Behavior Checklists.docx [file mep_2374-8265.11414-s001.zip › H. Empathy Session 2 Student Handout.docx]

We are going to watch some videos of simulated patient encounters. As we view them, I would like you to first remember the arts observation strategies we have learned and the questions we asked ourselves last week as we viewed paintings together.

Strategy 1: the Five Question Protocol (from the University of Rochester)

- What do you see?
- Does this remind you of anything?
- What is the story?
- What information would confirm that story?
- What did you observe about yourself?

Strategy 2: Visual Thinking Strategies

- What is going on in this picture?
- What do you see that makes you say that?
- What more can we find?

Strategy 3: Inquiry-Based looking

- What is going on in this picture?
- What do you see that makes you say that?
- What more can we find?

Strategy 4: Denotations/Connotations

- What do you see?
- What does that mean?

Questions to think about:

1. How do contextual factors influence what we observe and how we interpret it?
2. How did interactions between people influence how you interpreted this piece? How will that influence interpretations in the clinical setting?
3. How did you force yourself to see more than what stood out initially?
4. What might influence your interpretations of denotations?

As you watch these videos, I would like you to take notes and consider how you would rate the physicians on their degree of interaction with the patient/families based on the following milestone rubric (note this scale’s ratings go from 1-6):

#### Empathy with Patients and Families

#### Makes derogatory remarks about patient and/or family

1. Does not acknowledge cues or statements of emotion
2. Minimally acknowledges cues or statements of emotion
3. Acknowledges cues or statements of emotion with general reassurance
4. Explores cues and statements of emotion
5. Discusses emotions and suffering while affirming patient’s and/or family’s experience
