# Supplementary material for: An Empathy and Arts Curriculum During a Pediatrics Clerkship: Impact on Student Empathy and Behavior
Source: MedEdPORTAL. 2024 Jul 12;20:11414. doi: 10.15766/mep_2374-8265.11414 (PMC11239799; doi:10.15766/mep_2374-8265.11414)
Supplement: Supplementary file 1 — Empathy Session 1.pptxEmpathy Session 1 Facilitator Guide.docxEmpathy Session 2.pptxEmpathy Session 2 Facilitator Guide.docxEmpathy Video 1.mp4Empathy Video 2.mp4Empathy Video 3.mp4Empathy Session 2 Student Handout.docxEmpathy Session 1 Evaluation Form.docxEmpathy Session 2 Evaluation Form.docxToronto Empathy Questionnaire.docxEmpathy Behavior Checklists.docx [file mep_2374-8265.11414-s001.zip › K. Toronto Empathy Questionnaire.docx]

Below is a list of statements. Please read each statement carefully and rate how frequently you feel or act in the manner described. Circle your answer on the response form. There are no right or wrong answers or trick questions. Please answer each question as honestly as you can.

|  |  | Never | Rarely | Sometimes | Often | Always |
| --- | --- | --- | --- | --- | --- | --- |
| 1. | When someone else is feeling excited, I tend to get excited too | 0 | 1 | 2 | 3 | 4 |
| 2. | Other people's misfortunes do not disturb me a great deal | 0 | 1 | 2 | 3 | 4 |
| 3. | It upsets me to see someone being treated disrespectfully | 0 | 1 | 2 | 3 | 4 |
| 4. | I remain unaffected when someone close to me is happy | 0 | 1 | 2 | 3 | 4 |
| 5. | I enjoy making other people feel better | 0 | 1 | 2 | 3 | 4 |
| 6. | I have tender, concerned feelings for people less fortunate than me | 0 | 1 | 2 | 3 | 4 |
| 7. | When a friend starts to talk about his\her problems, I try to steer the conversation towards  something else | 0 | 1 | 2 | 3 | 4 |
| 8. | I can tell when others are sad even when they do not say anything | 0 | 1 | 2 | 3 | 4 |
| 9. | I find that I am "in tune" with other people's moods | 0 | 1 | 2 | 3 | 4 |
| 10. | I do not feel sympathy for people who cause their own serious illnesses | 0 | 1 | 2 | 3 | 4 |
| 11. | I become irritated when someone cries | 0 | 1 | 2 | 3 | 4 |
| 12. | I am not really interested in how other people feel | 0 | 1 | 2 | 3 | 4 |
| 13. | I get a strong urge to help when I see someone who is upset | 0 | 1 | 2 | 3 | 4 |
| 14. | When I see someone being treated unfairly, I do not feel very much pity for them | 0 | 1 | 2 | 3 | 4 |
| 15. | I find it silly for people to cry out of happiness | 0 | 1 | 2 | 3 | 4 |
| 16. | When I see someone being taken advantage of, I feel kind of protective towards him\her | 0 | 1 | 2 | 3 | 4 |

From: Spreng RN, McKinnon MC, Mar RA, Levine B. The Toronto Empathy Questionnaire: scale development and initial validation of a factor-analytic solution to multiple empathy measures. *J Pers Assess*. 2009 Jan;91(1):62-71. doi: 10.1080/00223890802484381
