# Supplementary material for: An Empathy and Arts Curriculum During a Pediatrics Clerkship: Impact on Student Empathy and Behavior
Source: MedEdPORTAL. 2024 Jul 12;20:11414. doi: 10.15766/mep_2374-8265.11414 (PMC11239799; doi:10.15766/mep_2374-8265.11414)
Supplement: Supplementary file 1 — Empathy Session 1.pptxEmpathy Session 1 Facilitator Guide.docxEmpathy Session 2.pptxEmpathy Session 2 Facilitator Guide.docxEmpathy Video 1.mp4Empathy Video 2.mp4Empathy Video 3.mp4Empathy Session 2 Student Handout.docxEmpathy Session 1 Evaluation Form.docxEmpathy Session 2 Evaluation Form.docxToronto Empathy Questionnaire.docxEmpathy Behavior Checklists.docx [file mep_2374-8265.11414-s001.zip › L. Empathy Behavior Checklists.docx]

**Empathy Behavior Checklist: Student**

After a recent clinical encounter, you requested the patient/family complete a survey on your bedside interactions. Using that same clinical encounter, please complete the survey below (using your unique identifier: day of birth + birth city), which is estimated to take approximately 5-10 minutes.

1. Please list your unique identifier (birth day + birth city): ______________

2. Today’s visit date:

| Question | Often | Sometimes | Not at all |
| --- | --- | --- | --- |
| 3.During this encounter, you asked open-ended questions (Example: questions begin with “what” as in “What symptoms prompted you to bring your child to the hospital?”) |  |  |  |
| 4.During this encounter, you demonstrated active listening. (Example: did not interrupt, you paid close attention to what the patient/family was saying.) |  |  |  |
| 5.During this encounter, you demonstrated empathetic physical body language (Example: sat at the patient/family level, faced the patient/family, leaned in/nodded while listening.) |  |  |  |
| 6. During this encounter, you demonstrated empathetic eye contact (Example: eyes on patient/family during conversation, minimal note taking/referencing.) |  |  |  |
| 7. During this encounter, you demonstrated empathetic hand gestures (Example: appropriate touches on the shoulder or hand to show concern.) |  |  |  |
| 8. During this encounter, you demonstrated empathetic conversational skills (Example: thoughtful tone, volume, cadence of speech.) |  |  |  |
| 9. During this encounter, you explored more about the patient/family’s experience (Example: you used language such as: “tell me more”, “go on”, or “what happened next?”) |  |  |  |
| 10. During this encounter, you identified and named emotions (Example: you used language such as: “this must feel frustrating” or “I wonder if you feel frustrated?”) |  |  |  |
| 11. During this encounter, you demonstrated understanding of emotions (Example: you used language such as “I can see how important this is to you.” or “This seems to be affecting you so much.”) |  |  |  |
| 12. During this encounter, you demonstrated respecting language (Example: you used language such as “You did the right thing by coming in today.” or “I’m impressed by all that you’ve done to manage your illness thus far.”) |  |  |  |
| 13.During this encounter, you used supporting language? (Example: you used language such as: “We are here to help you.” or “We will work hard to make sure your pain is well-controlled.”) |  |  |  |
| 14.Did you express concern directly to the child? (Example: to them, you asked “It looks like you don’t feel very good.” or “I’m sorry you’re feeling like this.”) |  |  |  |

15.Based on your encounter, please choose which description best describes your interaction with the patient/family.

a) makes derogatory remarks about patient/family

b) does not acknowledge cues or statements of emotions

c) minimally acknowledges cues or statements of emotions

d) acknowledges cues or statements of emotion with general reassurance

e) explores cues and statements of emotion

f) discusses emotions while affirming patient/family’s experience

16. During this encounter, did you do anything else (not mentioned above) to affect your bedside interactions with relation to empathy. If so, please describe in detail.

Thank you for filling out this survey. Have a nice day.

**Empathy Behavior Checklist: Patient and Family**

We’re studying patient perceptions of medical student empathy during their time on the Pediatrics clerkship.

Empathy is defined as “the ability to understand and share the feelings of another.”

We invite you to fill out this survey. It will take about 5-10 minutes to do so. Please share your thoughts about this student (using their unique identifier they will provide you: day of birth + birth city) on your most recent encounter with them.

Thank you so much for your participation.

1. Please list the unique identifier (birth day + birth city) of your student: ______________

2. Today’s visit date: ______________

| Question | Often | Sometimes | Not at all |
| --- | --- | --- | --- |
| 3.Did the student ask open-ended questions? (Example: They started their questions with “what” as in “What symptoms prompted you to bring your child to the hospital?”) |  |  |  |
| 4.Did the student actively listen to your story? (Example: They did not interrupt or hurry you and paid close attention to what you were saying.) |  |  |  |
| 5.Did the student sit at your level? |  |  |  |
| 6.Did the student face you and your child as they spoke to you? |  |  |  |
| 7.Did the student lean in to listen or nod while listening? |  |  |  |
| 8.Did the student make good eye contact? (Example: They kept their eyes on you while talking and listening, minimal note taking/looking at a computer.) |  |  |  |
| 9.Did the student use appropriate touches on the shoulder or hand to show concern? |  |  |  |
| 10.Did the student use thoughtful tone, volume, and timing in their speech? |  |  |  |
| 11.Did the student explore more about your child’s and your family’s experience? (Example: They used language such as: “tell me more”, “go on”, or “what happened next?”) |  |  |  |
| 12.Did the student identify and name emotions? (Example: They used language such as: “This must feel frustrating” or “I wonder if you feel frustrated?”) |  |  |  |
| 13.Did the student demonstrate understanding of emotions (Example: They used language such as “I can see how important this is to you.” or “This seems to be affecting you so much.”) |  |  |  |
| 14.Did the student use respecting language? (Example: They used language such as “You did the right thing by coming in today.” or “I’m impressed by all that you’ve done to manage your illness thus far.”) |  |  |  |
| 15.Did the student use supporting language? (Example: They used language such as: “We are here to help you.” Or “We will work hard to make sure your pain is well-controlled.”) |  |  |  |
| 16.Did the student express concern directly to your child? (Example: “It looks like you don’t feel very good.” Or “I’m sorry you’re feeling like this.”) |  |  |  |
| 17.Please choose which phrase best describes how the student interacted with you and your child. | The student made negative or disrespectful comments. | The student paid attention to some of the emotions you shared and responded to them with general reassurance. | The student talked about your feelings and affirmed your experience. |

18. Did the student do anything else (not mentioned above) to affect how you viewed their empathy? If so, please describe in detail. __________________________________________________________________________________________________

Thank you for filling out this survey. Have a nice day.
