# Supplementary material for: An Empathy and Arts Curriculum During a Pediatrics Clerkship: Impact on Student Empathy and Behavior
Source: MedEdPORTAL. 2024 Jul 12;20:11414. doi: 10.15766/mep_2374-8265.11414 (PMC11239799; doi:10.15766/mep_2374-8265.11414)
Supplement: Supplementary file 1 — Empathy Session 1.pptxEmpathy Session 1 Facilitator Guide.docxEmpathy Session 2.pptxEmpathy Session 2 Facilitator Guide.docxEmpathy Video 1.mp4Empathy Video 2.mp4Empathy Video 3.mp4Empathy Session 2 Student Handout.docxEmpathy Session 1 Evaluation Form.docxEmpathy Session 2 Evaluation Form.docxToronto Empathy Questionnaire.docxEmpathy Behavior Checklists.docx [file mep_2374-8265.11414-s001.zip › D. Empathy Session 2 Facilitator Guide.docx]

**Empathy Session 2: Facilitator Guide/Outline**

| **Session Objectives** | **Session Timeline** |
| --- | --- |
| List at least 5 behaviors that convey empathy at the bedside | 0-15min: Introduction/small group sharing (welcome/review of objectives/review of empathy components/shared witnessed emotions) |
| Describe emotions within a patient encounter utilizing a video of a simulated one | 15-20min: Review of techniques/priming, instruction for videos |
| Appraise physician behaviors during a patient encounter and describe methods by which empathy may be relayed better | 20-50min: Videos/small group discussion (10min/video) |
|  | 50-60min: Review of empathetic techniques/conclusions |

**Introduction: (15 minutes, slides 1-4)**

Thank you all for coming today. I hope we can continue our work from last week.

Let us quickly review our objectives for this time together. By the end of this session, we want to…

Objective1: List at least 5 behaviors that convey empathy at the bedside

Objective 2: Describe emotions within a patient encounter utilizing a video of a simulated one

Objective 3: Appraise physician behaviors during a patient encounter and describe methods by which empathy may be relayed better

Let’s remember the three components to empathy:

1. Emotion recognition: the cognitive ability to infer what another is feeling
2. Perspective taking: the ability to experience another person’s emotions
3. Affective response: responding with compassion/concern

We covered part 1 last week with learning those arts observation techniques to recognize emotions at the bedside. For part 2 in this, the perspective taking, literature suggests this comes from building our experiences, learning from different people, reading, and immersing ourselves in stories that are not just the ones we are the narrator within, and I think we tried practicing that last week as well when we applied those observation techniques to paintings and hearing each other’s thoughts on what we saw. Today we are going to talk a little bit more about part 3, that affective response that comes after recognizing an emotion.

First, let’s talk about your encounters, what are some empathetic behaviors you have witnessed or that you have used in your clinical time? Anyone want to begin by sharing an example from their time in the clinic? One of the important things about empathy is that it is not a prescription – it comes in many forms – through actions/behaviors/speech/timing – and you all really highlighted that in your sharing of prior experiences.

**Priming/Overview (3 minutes, slides 5/6)**

We’re going to watch some patient encounters now. I want you to utilize some of the techniques we reviewed last week when looking at art pieces as you watch this. Let’s quickly review them.

Strategy 1: the Five Question Protocol

- What do you see?
- Does this remind you of anything?
- What is the story?
- What information would confirm that story?
- What did you observe about yourself

Strategy 2: Visual Thinking Strategies

- What is going on in this picture?
- What do you see that makes you say that?
- What more can we find?

Strategy 3: Inquiry-Based looking

- What is going on in this picture?
- What do you see that makes you say that?
- What more can we find?

Strategy 4: Denotations/Connotations

- What do you see?
- What does that mean?

We also talked about 4 questions to ask ourselves that may influence what we are observing and I want you to consider those as well.

1. How do contextual factors influence what we observe and how we interpret it?
2. How do interactions between people influence how we interpret a scenario?
3. How do we force ourselves to see more that what stands out initially?
4. What might influence our interpretation of denotations?

As we watch these videos, I encourage you to take notes on what you see during this interaction in the handout provided (Appendix H). How do you think the patient/family is feeling? How do you think the provider does responding to those feelings? I want you to think about what kind of empathetic rating you would give this encounter, why, and what could be done better from a standpoint of empathy.

**Discontinue PowerPoint and pull up videos (10 minutes on each video/discussion)**

**Video 1:**

**Setting:** You are an overnight resident, admitting a patient at 1am. It has been a busy shift thusfar and this is your 5^th^ admission. You have just been paged by your senior that another one is about to come your way.

The signout you received from the ED is as follows: Emma Smith is a former 33 week premature, now 5 week old infant, presenting with fever to 101.5 at home. At her local ED, a full sepsis workup was attempted and both LP and urinalysis were unable to be obtained despite two attempts at each. CBC and blood cultures were obtained, she was given a dose of ceftriaxone and transferred to your hospital for further care.

**Video 2:**

**Setting:** You are a 3^rd^ year resident and just came back from vacation to your outpatient continuity clinic. Your flight yesterday was delayed and you didn’t get in till 2am.

You look at the patient chart in front of you; this is a 3 day old newborn baby recently discharged from the newborn nursery and here for routine follow up. You note that the baby’s weight is 11% down from birthweight and upon entering the room you can tell that the baby has jaundice through his chest.

**Video 3:**

**Setting:** You are working in the ED and see a 2 year old patient with a chief complaint of limp. His father is a nurse who works on the hematology/oncology floor of the Children’s Hospital, though you do not know him professionally. The nurse flags you down before you enter the room to tell you that the father has been very demanding, constantly asking that a battery of labs be performed and requesting an MRI as opposed to the xrays that have just been completed.

Questions to pose after each video (with some possible responses below).

1. What emotions did you note?: How did you notice those? How do contextual factors influence what we observe and how we interpret it? What is the story? Did you think to expect something and try and look for that?)
   1. Video 1: fatigue, isolation, worry, frustration
   2. Video 2: excitement, nervousness, confusion
   3. Video 3: worry, anger
2. What did you rate this encounter on your empathy scale? (How did you force yourself to see more than what stood out initially?)
3. In reviewing through the scenario, what are some things the physician could have done to express empathy better? (How did interactions between people influence how you interpreted this piece? How will that influence interpretations in the clinical setting?)
   1. Video 1: better eye contact, avoiding standing behind the computer, not interrupting, active listening and observation, cadence of speech, using understanding/respecting language
   2. Video 2: using “what” language instead of “how” language, open-ended questions instead of “Have you…?” questions, naming of emotions with reassurance
   3. Video 3: keeping tone of voice and cadence similar even in moments when the doctor felt flustered, naming emotions with reassurance
4. What else in the background may have contributed to his ability/inability to demonstrate empathy at the bedside? (What might influence your interpretations of denotations?)
   1. Video 1: it has been a busy shift, 5^th^ admission, you might be frustrated and trying to just get through the day
   2. Video 2: fatigue from lack of sleep
   3. Video 3: preconception of father being difficult after nursing signout

**Conclusions/Wrap-up: slides 7-12**

Discuss techniques/tips to consider when providing empathy at the bedside. Many will have been identified by students as they discuss the videos.

Here are some to review:

Open-ended questioning

Active listening and observing

Body language/positioning

Eye contact

Touch

Cadence/volume/tone of speech

Exploring the patient’s perspective

Naming emotions

Using understanding language

Using respecting language

Using supportive language

I wish statements

Thank you all for your participation!

I hope that this time together was valuable to you. And I hope that you will be able to utilize some of the things we talked about as you interact with patients during your rotation with us.

Before you leave, I’m going to give you a little bit of time to complete these anonymous surveys of today’s session. Please just drop them face-down on the chair in the back of the room. (**slide 13)**
