# Supplementary material for: An Empathy and Arts Curriculum During a Pediatrics Clerkship: Impact on Student Empathy and Behavior
Source: MedEdPORTAL. 2024 Jul 12;20:11414. doi: 10.15766/mep_2374-8265.11414 (PMC11239799; doi:10.15766/mep_2374-8265.11414)
Supplement: Supplementary file 1 — Empathy Session 1.pptxEmpathy Session 1 Facilitator Guide.docxEmpathy Session 2.pptxEmpathy Session 2 Facilitator Guide.docxEmpathy Video 1.mp4Empathy Video 2.mp4Empathy Video 3.mp4Empathy Session 2 Student Handout.docxEmpathy Session 1 Evaluation Form.docxEmpathy Session 2 Evaluation Form.docxToronto Empathy Questionnaire.docxEmpathy Behavior Checklists.docx [file mep_2374-8265.11414-s001.zip › I. Empathy Session 1 Evaluation Form.docx]

**Empathy Session #1 Evaluation Form**

Objectives:

- Identify emotions in hospitalized patients
- Describe 1 of 4 strategies for arts observation
- Apply learned strategies to discuss visual arts mediums
- Choose one particular observation strategy to utilize in patient care

The objectives of this session were met.

| Strongly agree | Agree | Neutral | Disagree | Strongly disagree |
| --- | --- | --- | --- | --- |
|  |  |  |  |  |

Learning arts observation strategies provides helpful observation tools to apply clinically at the bedside.

| Strongly agree | Agree | Neutral | Disagree | Strongly disagree |
| --- | --- | --- | --- | --- |
|  |  |  |  |  |

Practicing the arts observation strategies we learned makes me feel more comfortable utilizing them clinically at the bedside.

| Strongly agree | Agree | Neutral | Disagree | Strongly disagree |
| --- | --- | --- | --- | --- |
|  |  |  |  |  |

The facilitator engaged the group during this session.

| Strongly agree | Agree | Neutral | Disagree | Strongly disagree |
| --- | --- | --- | --- | --- |
|  |  |  |  |  |

Your written feedback for the following questions is greatly appreciated!

1. Is there an observation strategy you already use that has helped you in clinical practice?
2. Is there an observation strategy you are most likely to adopt and utilize moving forwards?

1. What from this session was most meaningful to you?
2. What about this session can be improved moving forwards?
