# Supplementary material for: An Empathy and Arts Curriculum During a Pediatrics Clerkship: Impact on Student Empathy and Behavior
Source: MedEdPORTAL. 2024 Jul 12;20:11414. doi: 10.15766/mep_2374-8265.11414 (PMC11239799; doi:10.15766/mep_2374-8265.11414)
Supplement: Supplementary file 1 — Empathy Session 1.pptxEmpathy Session 1 Facilitator Guide.docxEmpathy Session 2.pptxEmpathy Session 2 Facilitator Guide.docxEmpathy Video 1.mp4Empathy Video 2.mp4Empathy Video 3.mp4Empathy Session 2 Student Handout.docxEmpathy Session 1 Evaluation Form.docxEmpathy Session 2 Evaluation Form.docxToronto Empathy Questionnaire.docxEmpathy Behavior Checklists.docx [file mep_2374-8265.11414-s001.zip › J. Empathy Session 2 Evaluation Form.docx]

**Empathy Session #2 Evaluation Form**

Objectives: Utilizing a video of a simulated patient encounter,

1. Describe emotions within a patient encounter.
2. List at least 5 behaviors that convey empathy at the bedside
3. Appraise physician behaviors during a patient encounter and describe methods by which empathy may be better relayed

The objectives of this session were met.

| Strongly agree | Agree | Neutral | Disagree | Strongly disagree |
| --- | --- | --- | --- | --- |
|  |  |  |  |  |

Learning arts observation strategies provides helpful observation tools to apply clinically at the bedside.

| Strongly agree | Agree | Neutral | Disagree | Strongly disagree |
| --- | --- | --- | --- | --- |
|  |  |  |  |  |

Practicing the arts observation strategies we learned makes me feel more comfortable utililizing them clinically at the bedside.

| Strongly agree | Agree | Neutral | Disagree | Strongly disagree |
| --- | --- | --- | --- | --- |
|  |  |  |  |  |

I learned new empathetic techniques or this session reinforced ones I already use in clinical care.

| Strongly agree | Agree | Neutral | Disagree | Strongly disagree |
| --- | --- | --- | --- | --- |
|  |  |  |  |  |

The facilitator engaged the group during this session.

| Strongly agree | Agree | Neutral | Disagree | Strongly disagree |
| --- | --- | --- | --- | --- |
|  |  |  |  |  |

Your written feedback for the following questions is greatly appreciated!

1. Are there common empathetic responses you use in patient care? If so, what are they?
2. Did you learn a new empathetic response in this session to use in the future? If so, what was it?

1. What from this session was most meaningful to you?
2. What about this session can be improved moving forwards?
